# Scalable Multistep Roll‐to‐Roll Printing of Multifunctional and Robust Reentrant Microcavity Surfaces via a Wetting‐Induced Process

**DOI:** 10.1002/adma.202411064

**Published:** 2024-11-21

**Authors:** Su Hyun Choi, Seungwoo Shin, Woo Young Kim, Je Min Lee, Seo Rim Park, Hyuntae Kim, Kyoohee Woo, Sin Kwon, Nicholas X. Fang, Seok Kim, Young Tae Cho

**Affiliations:** ^1^ Department of Advanced Battery Manufacturing Systems Korea Institute of Machinery & Materials Daejeon 34103 South Korea; ^2^ Department of Smart Manufacturing Engineering Changwon National University Changwon 51140 South Korea; ^3^ Department of Mechanical Engineering The University of Hong Kong Hong Kong 999077 China; ^4^ Department of Mechanical Engineering Changwon National University Changwon 51140 South Korea

**Keywords:** matter‐loadable microcavity surfaces, multifunctional reentrant surfaces, multistep roll‐to‐roll printing, superior liquid repellency, wetting‐induced

## Abstract

Owing to their unique structural robustness, interconnected reentrant structures offer multifunctionality for various applications. a scalable multistep roll‐to‐roll printing method is proposed for fabricating reentrant microcavity surfaces, coined as wetting‐induced interconnected reentrant geometry (WING) process. The key to the proposed WING process is a highly reproducible reentrant structure formation controlled by the capillary action during contact between prefabricated microcavity structure and spray‐coated ultraviolet‐curable resins. It demonstrates the superior liquid repellency of the WING structures, which maintain large contact angles even with low‐surface‐tension liquids, and their robust capability to retain solid particles and liquids under external forces. In addition, the scalable and continuous fabrication approach addresses the limitations of existing methods, providing a cost‐effective and high‐throughput solution for creating multifunctional reentrant surfaces for anti‐icing, biofouling prevention, and particle capture.

## Graphical abstract

The multifunctionality of reentrant microcavity geometries for different materials is investigated, and a novel and efficient method for fabricating such geometry is proposed by analyzing capillary fluid behavior. This approach addresses the limitations of existing methods to provide a cost‐effective and high‐throughput solution for generating multifunctional reentrant surfaces, which is expected to be utilized in various fields.

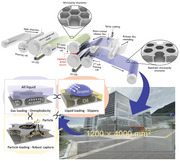

## 1 Introduction

Interconnected reentrant structures with mechanical robustness have evolved to impart multifunctionality to the outermost surfaces or skin of various living organisms, including springtail cuticles, diving beetles, citrus fruits, pitcher plant peristomes, and honeycombs. These structures facilitate adaptation and resilience in challenging environments. For instance, the interconnected ridges and granules with overhangs observed in springtails provide long‐term plastron stability, resulting in omniphobicity that protects their skin respiration from suffocation caused by complete wetting.^[^ ^1^ ^]^ Similarly, the interconnected microcavities in citrus fruits stably maintain citrus oils, which are known for their antimicrobial activity and toxicity to arthropods, thus protecting fruits and seeds from infection and predation.^[^ ^2^ ^]^ Inspired by honeycomb architectures, jar‐like reentrant honeycomb microstructures have proven effective for loading biological particles, with applications in drug or cell delivery and lossless cell seeding.^[^ ^3^ ^]^ The unique ability of reentrant structures to load multiphase matter is attributed to their concave topographic characteristics from top to bottom, which allow them to take various shapes, including trapezoidal, undercut, mushroom, and overhang. Consequently, many researchers have explored the engineering of functional surfaces by leveraging rationally designed reentrant structures inspired by nature. With the advancement of manufacturing techniques such as photolithography,^[^ ^4^, ^5^, ^6^ ^]^ imprint lithography,^[^ ^1^, ^7^, ^8^ ^]^ soft replication,^[^ ^9^, ^10^ ^]^ 3D printing,^[^ ^11^, ^12^, ^13^ ^]^ self‐assembly, microfluidics, templating, and capillary wetting,^[^ ^14^, ^15^, ^16^, ^17^, ^18^ ^]^ intricate reentrant geometric structures have exhibited superior functionality with regard to liquid repellency, adaptive adhesion of solid‐phase materials, and directional fluid transport. These capabilities have drawn considerable attention from academia and have been exploited in practical applications such as anti‐icing,^[^ ^19^, ^20^, ^21^, ^22^ ^]^ biofouling prevention,^[^ ^23^, ^24^, ^25^, ^26^ ^]^ and particle‐capturing surfaces.^[^ ^27^, ^28^ ^]^

However, a primary challenge in realizing reentrant‐structured surfaces is achieving high‐throughput large‐scale manufacturing for practical applications. Conventional lithography‐based techniques require sophisticated equipment, reentrant molds, and multiple steps to form reentrant structures,^[^ ^1^, ^4^, ^5^, ^6^, ^7^, ^8^, ^9^, ^10^, ^29^, ^30^ ^]^ which do not facilitate continuous large‐area fabrication. Although self‐assembly^[^ ^14^ ^]^ and microfluidics‐based approaches^[^ ^15^, ^16^ ^]^ are simple and cost‐effective, controlling the morphology of reentrant structures over large areas remains challenging. 3D‐printing technologies^[^ ^11^, ^12^, ^13^ ^]^ have printed complex, hierarchical reentrant structures with nanoscale or microscale resolutions; however, they still suffer from limited printable areas. Additionally, improving the mechanical robustness of reentrant structures under shear forces, potentially through structural interconnectivity, presents further challenges for large‐area high‐throughput manufacturing.^[^ ^31^, ^32^ ^]^

**Figure 1:**
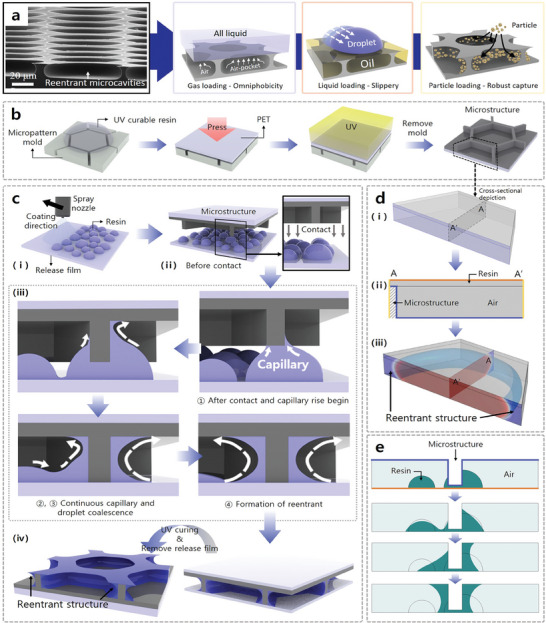
a) Fabricated reentrant microcavities and 3D renderings indicating the functional advantages of these structures. b) Schematic of the fabrication procedure for microcavity structures using UV‐nanoimprint lithography (NIL). c) Reentrant microcavities created via the wetting‐induced capillary phenomena of UV‐curable viscous liquid resins (WING process). d,e) Numerical analysis results for the capillary action involved in fabricating a reentrant structure, assuming that the resin was ideally coated uniformly and as droplets through spray coating (yellow line: symmetric condition; blue line: contact angle of resin on the microstructured surface; orange line: surface energy of release film).

In this paper, we propose a facile method for fabricating interconnected reentrant geometries over large areas using the wetting‐induced capillary phenomenon of ultraviolet (UV)‐curable viscous liquid resins. The proposed wetting‐induced interconnected reentrant geometry (WING) process allows stable loading of multiphase materials such as gases, solid particles, and liquids, making it suitable for various applications (**Figure** **1**a). Additionally, we demonstrated the mechanical robustness of WING structures and their compatibility with scalable and continuous roll‐to‐roll (R2R) manufacturing processes for interconnected reentrant‐structured surfaces, verifying their commercial applications.

## 2 Results and Discussion

The reentrant structure produced by this process exhibited high durability against external forces (Figure S1, Supporting Information) and has an interconnected microcavity architecture designed to enhance various surface functionalities by maximizing the material‐loading capacity (Figure 1a). These microcavities serve as pockets for capturing diverse substances. The reentrant shape with its horizontal top structure imparts an enclosed nature to the microstructure, enhancing its material collection capabilities. This feature renders it suitable for capturing substances in solid, liquid, and gaseous phases. For instance, when gaseous air is captured, a strong air‐pocket effect is created, resulting in an omniphobic surface with high liquid repellency irrespective of the liquid type. Moreover, loading a liquid such as a lubricant can extend the lifespan of slippery surfaces by preventing the depletion of lubricant oil, which is crucial for manufacturing bioinspired slippery surfaces. Additionally, capturing solid particles, enabling functions such as storage and preventing the spread of specific particles. Figure 1b,c presents a detailed schematic of the production of interconnected reentrant structures via the wetting‐induced wrapping of UV‐curable viscous resins around interconnected microcavity structures. First, interconnected microcavity structures were prepared using UV nanoimprint lithography (UV‐NIL) as described in our previous works (Figure 1b).^[^ ^27^, ^33^ ^]^ Subsequently, UV‐curable viscous resins were coated onto the release films (Figure 1c (i)). The prefabricated interconnected microcavity structures were gently brought into contact with the resin‐coated release film (Figure 1c (ii)). Upon contact, the viscous liquid resin spontaneously wetted the sidewalls of the microcavity structures owing to capillary forces, resulting in the formation of an interconnected reentrant geometry (Figure 1c (iii)). After UV exposure, the crosslinked interconnected reentrant geometry detached from the release film, completing the fabrication of the proposed WING, as shown in Figure 1c (iv). In this study, the selection of an appropriate coating method to effectively induce the wetting of UV‐curable liquid resins for WING formation was critical particularly considering integration with a continuous R2R‐based manufacturing system. Among the various coating methods, such as slot‐die coating,^[^ ^34^ ^]^ gravure roll coating,^[^ ^35^ ^]^ bar coating,^[^ ^36^ ^]^ and spray coating,^[^ ^37^, ^38^ ^]^ we selected spray coating because of its alignment with criteria for throughput, scalability, cost‐effectiveness, and system integration. In contrast to other coating technologies, spray coatings form microscale droplets on substrates. To assess the feasibility of spray coating using the proposed WING process, we conducted a numerical analysis (Figure 1d,e). These analyses were performed considering the surface energy at the interface of each material as a variable. Figure 1d presents a 3D numerical simulation designed to verify whether the UV‐curable resin forms a reentrant structure via capillary action when in contact with the microstructure. The simulation model is a schematic cross‐sectional representation of a unit cell from the interconnected hexagonal pattern (Figure 1d (i)), with boundary conditions as depicted in Figure 1d (ii). As shown in Figure 1d (iii), the analysis demonstrates that generated capillary action along the sides of the microstructure facilitates the formation of a reentrant structure. Figure 1e illustrates a separate numerical analysis aimed at confirming the formation of a reentrant structure when the UV‐curable resin, applied in droplet form, interacts with the microstructure. The results indicate that this structure is formed through the same capillary mechanism as described in the schematic in Figure 1c (iii). Based on these numerical results, we inferred the behavior of capillary action in relation to the surface energy of different materials, as shown in Figure S2 (Supporting Information). In summary, among the three materials used in the WING process, the WING structure is formed when the UV‐curable resin is coated on a release film with a lower surface energy than the surface energy of the microstructure. This conclusion is corroborated by experimental results using substrates with different surface energies, as depicted in Figure S3 (Supporting Information).

Reentrant structures with these characteristics have traditionally been manufactured through complex processes such as photolithography and etching. However, such structures can also be fabricated using UV‐NIL combined with a simple process that leverages the capillary phenomenon, as shown in Figure 1b,c. This method relies on the capillary action resulting from the interaction between the surface energy of the microstructure and the surface energy of the uncured resin. Furthermore, during the WING process, an excessive resin coating thickness can cause capillary action to fill the microcavities, resulting in a single thick membrane rather than a reentrant structure. Therefore, it is crucial to apply an appropriate resin coating thickness based on the microstructural dimensions used in the WING process. For the microstructure utilized in this study, a reentrant structure was confirmed to form when the resin coating thickness was <6 µm (Supporting Information 3). Moreover, it was confirmed that when the resin coating thickness is less than 6 µm, the reentrant structure is formed only at the upper part of the microstructure, or the hyperbolic geometry is formed (Figure S6, Supporting Information). Considering the durability of the reentrant structure, the hyperbolic reentrant geometry is considered to be advantageous, but excessive expansion of the reentrant structure may deteriorate the loading performance for various substances due to the increase in surface area and the reduction of the internal microcavity.

The shape of the microstructure used in the WING process can affect the reentrant geometry and the loading performance of various materials. Therefore, an experiment was conducted to analyze the effect of the geometry of the microstructure. By setting the length of one side as a geometry variable of the hexagonal pattern, as shown in Figure S7a–e (Supporting Information), a total of four shapes of hexagonal reentrant microcavity structures were manufactured using the WING process, and the solid fraction (*f_sl_*) was calculated to analyze the liquid repellency according to it. Here, the solid fraction is a key index for analyzing the liquid repellency of the surface with the microstructure engraved, which is the ratio of the solid surface where the droplet comes into contact with the surface with the microstructure engraved. Through the experimental results, we confirmed that as the length of one side of the microstructure increases, the solid fraction decreases, and the liquid repellency of the reentrant structure tends to improve.

**Figure 2:**
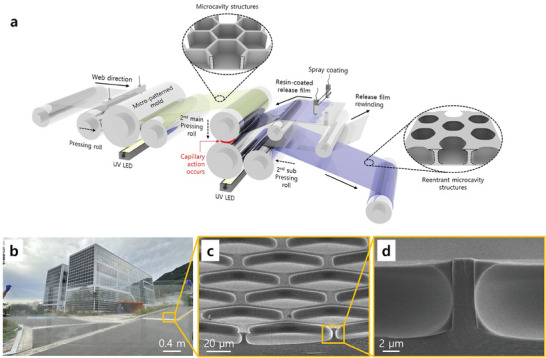
Scalable and continuous manufacturing of reentrant microcavity surfaces via multistep R2R printing. a) Schematic of the multistep R2R system (a photograph is presented in Figure S9, Supporting Information). These images were used to fabricate WING from a UV‐curable imprinting resin in an R2R process. b) Large‐area multifunctional reentrant microcavity surfaces manufactured via the high‐throughput scalable R2R imprinting process. c,d) Scanning electron microscopy (SEM) images of the reentrant microcavity surfaces.

Furthermore, the WING process, which integrates UV‐NIL and spray‐coating technologies, can be incorporated into R2R continuous printing for high‐throughput production. **Figure** **2**a presents a schematic of the multistep UV‐R2R printing process used to continuously produce a large‐scale WING. The UV‐R2R process comprises two stages. The first stage involves the manufacturing of microstructured surfaces using R2R nanoimprint lithography. In the second stage, a liquid UV‐curing resin is coated onto a specific substrate, which is then brought into contact with the prefabricated microstructure to induce capillary action. When the resin‐coated substrate has a surface energy lower than the interfacial free energy of the microstructure and the resin, large‐area reentrant surfaces are formed upon release of the two films after UV curing (Supporting Information 2). By developing equipment capable of performing this process (Figure S9, Supporting Information), large‐area surfaces with uniform reentrant structures having dimensions of 1200 × 4000 mm^2^ were fabricated (Figure 2b). These advanced results were achieved based on the experimental verification of the resin coating thickness uniformity using the spray coating technique shown in Figure S10 (Supporting Information), which was conducted for process optimization of the R2R WING process, and the analysis of the uniformity of the reentrant structures formed under the R2R WING process conditions shown in Figure S13 (Supporting Information).

**Figure 3:**
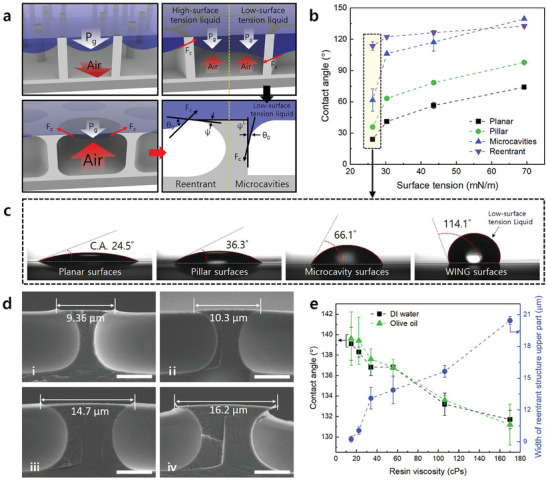
a) Schematics of the air‐loading tendencies of various microstructures. b) Static contact angle measurement results according to the surface tension of the droplet and c) contact angle measurement result images (surface tension = 26.3 mn m^−1^). d) SEM images of reentrant microcavities fabricated using UV‐curable resins with various viscosities (i. 22 cPs, ii. 34 cPs, iii. 56 cPs, iv. 106 cPs). The white scale bars represent 5 µm. e) Improved liquid repellency of reentrant microcavity surfaces manufactured with various viscosities of the UV‐curable resin.

Among the functions of the reentrant structure, the most extensively researched is omniphobicity, which has been interpreted according to contact‐angle theory. This theory explains the repellency of a droplet in contact with a surface by considering the energy acting at the interfaces between the solid, liquid, and gas phases. The Cassie–Baxter model—the most widely applied contact‐angle theory for uneven surfaces—is based on the air‐pocket effect, in which air trapped between microstructures supports droplets in contact with the surface. However, the Cassie state cannot be indefinitely maintained on a surface with an engraved microstructure. Because of the capillary phenomenon, which is influenced by the correlation between the droplet pressure continuously applied by gravity and the interfacial energy, the Cassie state eventually transitions to the Wenzel state, which is a fully wetted state. Krupenkin et al. highlighted the potential for reversible transitions between the Wenzel and Cassie states.^[^ ^39^ ^]^ Tuteja et al. revealed that for the Cassie–Baxter contact‐angle theory to be satisfied on a surface with a microstructure rather than a reentrant structure, the condition of *θ_0_* ≥ 90° must be met.^[^ ^40^ ^]^ This condition is achievable when the surface energy of the solid constituting the surface is low or when the surface energy of the contacting liquid is high. However, when a liquid with low surface tension contacts the surface, *θ_0_* becomes <90°, as shown in **Figure** **3**a, and the transition to the Wenzel state is accelerated. This phenomenon can be explained in terms of free energy acting at the material interface. The wetting of the side of the microstructure can be understood as a transition from a solid–gas interface to a solid–liquid interface due to liquid penetration. When the surface tension of the penetrating liquid is low, converting the solid–gas interface into a solid–liquid interface is energetically advantageous for maintaining a state of equilibrium. Consequently, the direction of the capillary force generated as the liquid wets the side of the microstructure is directed downward, and *θ_0_* falls below 90°, accelerating the transition to the Wenzel state. In contrast, on a surface with an engraved reentrant structure, the upper part of the structure has a double structure in the horizontal direction. Thus, the capillary force acts horizontally along the end of the reentrant structure. Additionally, owing to the strong air‐pocket effect, the transition from a solid–gas interface to a solid–liquid interface when the liquid penetrates is energetically disadvantageous for maintaining equilibrium. Hence, even if *θ_0_* < 90°, the surface has high liquid repellency. This phenomenon occurred regardless of whether the surface tension of the liquid was low or high. To verify the phenomenon, liquids with different surface tensions were used to measure the static contact angles of the surfaces according to their microstructures. As shown in Figure 3b,c, a contact angle of >130° was measured for a liquid with high surface tension in the microcavity structure. However, as the surface tension of the liquid decreases, the contact angle decreases. For a liquid with a surface tension of ≈26 mN m^−1^, the contact angle rapidly decreased to ≈60°. In contrast, on the surface with WING, a contact angle of more than 110° was measured, even for liquids with low surface tension. For a surface with an engraved pillar structure, the microstructure is open. Thus, air loading does not occur, resulting in a low contact angle as air escapes between the microstructures.

To further improve the liquid repellency of reentrant structures, which already demonstrate superior performance due to their inherent structural properties, it is essential to account for the surface area where the droplet interacts with the microstructure. Notably, since the reentrant structures produced through the WING process rely on capillary action, adjusting the process conditions to vary the dimensions of these structures inevitably alters the top width (surface area, solid fraction). In this regard, Kim et al. quantitatively analyzed the solid fraction of surfaces with different microstructures and established its correlation with liquid repellency.^[^ ^41^ ^]^ To reduce the surface area of the reentrant structure fabricated using the WING process, it is essential to analyze the mechanism of capillary action. For this purpose, a dimensionless coefficient called the capillary number was introduced. The capillary number represents the effect of the viscous force relative to the interfacial energy of the liquid and surface.^[^ ^42^, ^43^ ^]^

In other words, the effects of the capillary and viscous forces on the behavior of the fluid (UV‐curable resin) during capillary action can be analyzed. The analysis of the capillary number revealed that the viscous force had a more significant effect on the capillary fluid behavior during the WING process (Figure S7, Supporting Information). This was confirmed by conducting the WING process using UV‐curable resins of different viscosities. As shown in Figure 3d, the WING structure fabricated using low‐viscosity UV‐curable resin through the WING process exhibited a smaller top width. Furthermore, the 3D laser scanning of the top‐view images, presented in Figure S14 (Supporting Information), visually confirms the reduction in surface area of the reentrant structures.

The physical behavior of the WING structures, which varies depending on the viscosity of the UV‐curable resin described above, can be explained by summarizing the relationships in the Washburn equation (Equation 1) and the equations describing the volume change of the coated resin before and after capillary action (Equations 2 and 3),^[^ ^18^, ^44^ ^]^ leading to the formulation of Equation (4). In deriving these relationships, the shape of the capillary meniscus was simplified to a triangular form, and the hexagonal pattern was simplified to a circular pattern to analyze the volume change of the resin (Figure S15, Supporting Information).

(1) $$z\left(t\right)∼{\left(\frac{\gamma Dt}{2\eta }\right)}^{1/2}$$

(2) $$V_{ini}=\frac{\pi }{4}D^{2}\cdot t_{c}$$

(3) $$V_{aft}=\pi \cdot z\left(t\right)\left[D\cdot w\left(t\right)-\frac{4}{3}w{\left(t\right)}^{2}\right]$$

(4) $$V_{ini}\approx V_{aft}=\alpha \cdot {\frac{D}{4}}^{2}\cdot t_{c}\approx {\left(\frac{\gamma Dt}{2\eta }\right)}^{1/2}\cdot \left[D\cdot w\left(t\right)-\frac{4}{3}w{\left(t\right)}^{2}\right]$$

In the above equation, *z* represents the capillary rise height, while *γ*, *D*, *t*, and *η* correspond to the surface tension of the UV‐curable resin, the diameter of the cavity where capillary action occurs, the duration of the capillary action, and the viscosity of the UV‐curable resin, respectively. Additionally, *V_ini_* and *V_aft_* denote the volume of the UV‐curable resin before and after capillary action, while *t_c_* and *w* represent the coating thickness of the UV‐curable resin and the width of the upper part of the WING structure, respectively (Figure S15, Supporting Information). It should be noted that not all of the initially coated UV‐curable resin contributes to forming the capillary meniscus during the WING process. As shown in the 3D laser scanning images in Figure S16 (Supporting Information), residual resin remains in the center of the microcavity where the capillary meniscus has not formed. Therefore, a constant *α* was introduced into *V_ini_* to account for the volume of resin lost after forming the capillary meniscus.

In Equation (4), it can be seen that 𝑤 is proportional to *η* and *t_c_*. As shown in Figure 3e; Figure S6 (Supporting Information), it was confirmed that the top width of the reentrant structure fabricated via the WING process increased proportionally with the viscosity of the UV‐curable resin and the coating thickness. Notably, the WING surfaces manufactured using UV‐curable resin with a viscosity of 22 cPs exhibited high liquid repellency, with contact angles of ≈140° for both deionized (DI) water and olive oil. The top width of the theoretical reentrant structure, calculated using Equation (4), was compared with that of the reentrant structure fabricated through the actual WING process, and the same trend was observed, as shown in Figure S17 (Supporting Information).

In summary, it was confirmed that the changes in the reentrant geometry fabricated through the WING process, and thereby the surface liquid repellency, are related to *η* and *t_c_*.

Another approach to enhancing the liquid repellency of the WING structure is to coat the surface with silica nanoparticles.^[^ ^17^, ^22^ ^]^ After applying a silica coating to a large‐area WING structure film (1200 × 900 mm) fabricated via the R2R WING process, contact angles were measured using DI water and olive oil, yielding high liquid repellency values of 170.3° and 155.4°, respectively (Figure S18, Supporting Information). As shown in Table S5 (Supporting Information), these results demonstrate superior performance compared to previous studies.

**Figure 4:**
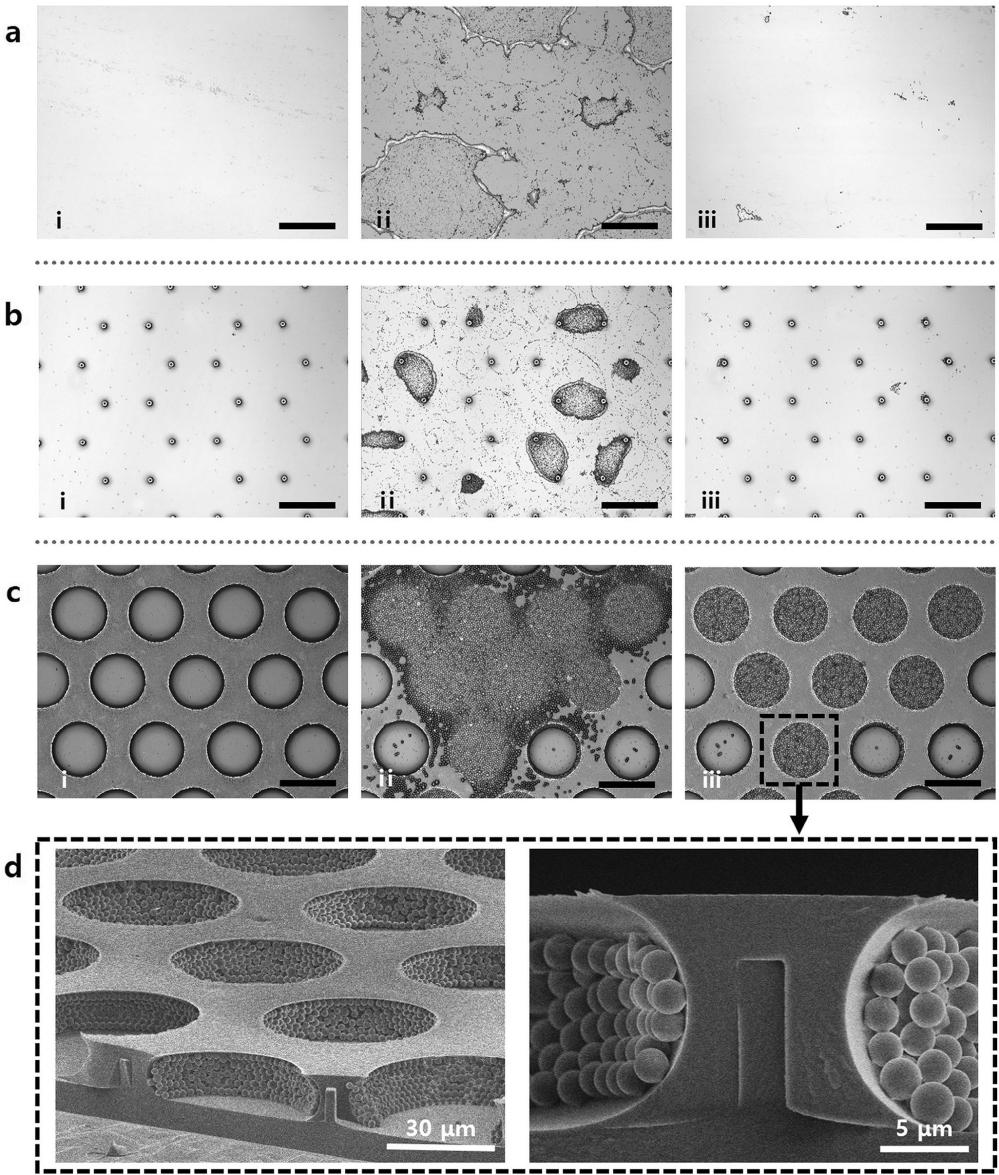
a–c) Surface morphology results from the particle‐loading test. (a) Planar surface, (b) pillar structure, and (c) reentrant microcavities at different stages: (i) before the particle‐loading test, (ii) after spraying particles, and (iii) after the tape peel‐off test. The black scale bars represent 50 µm. d) SEM images of microparticles captured within the reentrant microcavities.

The reentrant structure of the WING is expected to provide robust loading performance not only for air but also for materials in other phases. The loading of the solid materials was verified using a specific experimental procedure. The particles were loaded onto the surface by coating it with a solution of particles mixed with DI water at a predetermined ratio, wetting the surface, and allowing it to dry thoroughly. Subsequently, a tape peel‐off test was conducted to assess the robustness of the loaded particles (Figure S19, Supporting Information). The experimental results for the planar and pillar‐structured surfaces (**Figure** **4**a,b) confirmed that the particles were initially loaded onto the surface after the mixed solution was dried. However, after the tape peel‐off test, the particles were completely removed, indicating that the open structures lacked cavities for secure particle separation. In contrast, for the reentrant microcavity surfaces, the particles were observed to be loaded around the reentrant structure. After the tape peel‐off test, unlike the planar and pillar surfaces, the particles inside the reentrant microcavities remained firmly in place (Figure 4c). This robustness is attributed to the structural advantages of the reentrant microcavity, as previously described. The SEM image in Figure 4d shows the particles loaded inside the reentrant microcavity, indicating robust particle‐loading performance that resists external forces.

**Figure 5:**
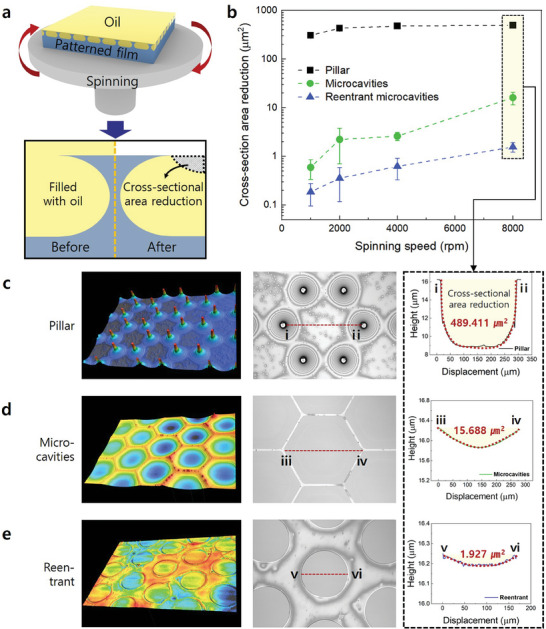
a) Schematic of spinning experiments for verifying the liquid‐loading performance. b) Results of spinning experiments (log scale). c–e) Surface morphology after spinning experiments and comparison of the cross‐sectional area reductions for different microstructures (spin rate = 8000 rpm).

Finally, liquid materials can also be effectively loaded using the WING. For liquids, penetration is challenging because of air pockets formed inside the WING. However, reversible conversion to the Wenzel state occurs because of the correlation between the free energies acting at the material interface. This transition is particularly accelerated for liquids with low surface tension, such as methanol (*γ_lv_* = 22.6 mN m^−1^), acetone (*γ_lv_* = 23.7 mN m^−1^), and hexane (*γ_lv_* = 18.4 mN m^−1^). The liquid loaded inside the reentrant microcavities was restrained from escaping because of the reentrant and enclosed structural characteristics. This was verified through a simple spinning experiment, as shown in **Figure** **5**a. After the microstructured surface was coated with silicone oil (*γ_lv_* ≈20.0 mN m^−1^) with low surface tension, sufficient waiting time was allowed for the transition to the Wenzel state. The liquid‐loading performance was compared by measuring the reduced cross‐sectional area after the spinning test, as illustrated in Figure 5a. Before the spinning test, it was assumed that the liquid was loaded to the same level as the height of the microstructures. All the loaded oil escaped even under low‐speed conditions in the case of the pillar structure (Figure 5c). This is because a cavity capable of trapping the liquid was not present, making it impossible to prevent oil escape, except for the oil distributed along the edge of the pillar, owing to capillary action. The spinning experiment utilized an outward centrifugal force. On surfaces with microcavities, the fine structure functions as a barrier, preventing the liquid from escaping due to the centrifugal force. This effect was verified by the results of spinning experiments on surfaces with microcavities and reentrant microcavities (Figure 5d,e). Although microcavities were formed on the surface, the upper part was completely open, causing liquid escape to rapidly increase under high‐speed spinning conditions. In contrast, owing to the structural advantages of the reentrant microcavities, liquid escape can be prevented even under high‐speed spinning conditions.

## 3 Conclusion

This work focused on the feasibility of large‐area wetting‐induced reentrant geometry (WING) process to leverage the multifunctionality of reentrant microcavities by leveraging their structural advantages. Our study revealed that an essential factor for successfully manufacturing reentrant structures is the correlation between the surface energy of each interface involved in the capillary action and the coating thickness of the UV‐curable resin. The advantageous shape of the reentrant microcavities produced through the WING process allows them to capture various materials in different phases. In experiments, reentrant microcavities exhibited superior liquid repellency to conventional microstructures, maintaining large contact angles even with low‐surface‐tension liquids. Furthermore, the robust particle‐loading capability of the reentrant structure was verified through tape peel‐off tests, in which particles remained firmly inside the microcavities, in contrast to planar and pillar surfaces. The liquid‐loading performance was tested via spinning experiments, in which the reentrant microcavities retained liquids under high‐speed conditions because of their enclosed structural nature. Overall, the WING process allows the fabrication of multifunctional reentrant microcavity surfaces with diverse applications. These surfaces offer groundbreaking opportunities by providing flexible solutions to unpredictable natural phenomena and addressing current limitations of scientific research and development. Because of their ability to capture and retain materials in different phases, coupled with their excellent liquid repellency, reentrant microcavity surfaces are a promising innovation in materials science and engineering.

## 4 Experimental Section

### Materials

Polyurethane‐acrylate type resins, MINS‐311RM (Minuta Technology Co., Ltd.), and low‐viscosity 311RM (Minuta Technology Co., Ltd.) were used to analyze the effect of resin viscosity on the WING process. UV‐curing resins were manufactured and mixed with MINS‐311RM and low‐viscosity 311RM at specific weight ratios (2:8, 4:6, 6:4, and 8:2). The two resins were mixed using a high‐speed digital stirrer (Daihan Scientific Co., Ltd., HS‐T) overnight at 200 rpm. A total of two substrates were used during the process, and a polyethylene terephthalate (PET) film (100 µm thick, SK Chemical Co., Ltd.) was used as the substrate on which micropatterns were formed. A CY801 film (38 µm thick, Kolon Industries) was used as the release film. Two types of particles (polystyrene microspheres (Fluo‐Spheres Caboxylate‐Modified Microspheres, 0.2 and 2 µm) were purchased from Thermo Fisher Scientific Co., Ltd. and used to conduct particle‐loading experiments. The particles were diluted with DI water to a concentration of ≈10^11^ particles Ml^−1^. Silicone oil (KF‐96, 10 cs, ShinEtsu Co., Ltd.) was used for the liquid‐loading experiments.

### Fabrication of Microstructures

The interconnected microstructure was designed to have a width of 5 µm, height of 10 µm, and array sidelength of 40, 60, 80, and 100 µm (Figure S20, Supporting Information). After producing a photomask with the designed pattern shape, a Si mold was produced on a Si wafer using photolithography and reactive ion etching. Subsequently, a film with an interconnected microstructure was produced using nanoimprint lithography. The microstructures fabricated using nanoimprint lithography had actual dimensions of 3–5 µm in width and 9 µm in height, with the sidelength matching the designed specifications.

### Measurements

Contact‐angle measurements were performed to analyze the liquid repellency of the surface engraved with reentrant microcavities. The liquids used were DI water and olive oil, and they were measured using a liquid‐droplet analysis tool (SmartDrop, Femtobiomed Co., Ltd.). The droplet volume used for these measurements was 10 µL. In addition, the same equipment was used to measure the physical properties (surface tension, viscosity) of uncured resin, and the conditions of the measurement environment were maintained at 22 °C (±3 °C) and 50% relative humidity (±5%). Each measurement was performed five times.

### Structural Characterization

Field‐emission SEM (S‐4800, Hitachi Co., Ltd.) and laser scanning confocal microscopy (VK‐X1000, Keyence Co., Ltd.) were used to analyze the microstructural characteristics of the microstructures fabricated through the process.

## Conflict of Interest

The authors declare no conflict of interest.

## Supporting information



Supporting Information

Supplemental Video 1

Supplemental Video 2

## Data Availability

The data that support the findings of this study are available from the corresponding author upon reasonable request.
