# Supplementary material for: Scalable Multistep Roll‐to‐Roll Printing of Multifunctional and Robust Reentrant Microcavity Surfaces via a Wetting‐Induced Process
Source: Adv Mater. 2024 Nov 21;37(5):2411064. doi: 10.1002/adma.202411064 (PMC11795719; doi:10.1002/adma.202411064)
Supplement: Supplementary file 1 — Supporting Information [file ADMA-37-2411064-s003.docx]

Supporting Information

**Scalable Multistep Roll-to-Roll Printing of Multifunctional and Robust Reentrant Microcavity Surfaces via a Wetting-Induced Process**

Su Hyun Choi, Seungwoo Shin, Woo Young Kim, Je Min Lee, Seo Rim Park, Hyuntae Kim, Kyoohee Woo, Sin Kwon, Nicholas X. Fang, Seok Kim*, and Young Tae Cho*

Supporting Information 1. Durability of WING structure against external forces

The mechanical stability of the WING structure was evaluated using a pencil hardness test, conducted according to the JIS K5600-5-4 standard, as shown in the schematic in **Figure S1a**. The WING film used for the test was a section of a large-area WING film, 1,200 mm in width, fabricated through the R2R WING process. The pencil lead was first sharpened to expose about 5 to 6 mm and then flattened at the tip. It was then brought into contact with the surface of the WING structure at a 45˚ angle and pressed with a load of 0.75 kgf. After preparing the test setup, the pencil hardness test was performed by moving the pencil horizontally over a distance of 20 mm at a speed of 1 mm/s, and the mechanical stability of the WING structure was evaluated using SEM imaging. As shown in Figure S1(b–d), the WING structure retained its reentrant shape at a hardness of 2 H, while localized scarring was observed at 3 H, and the structure fractured at 4 H. These results indicate that the WING structure on the large-area film exhibited an approximate hardness of 2.5 H.


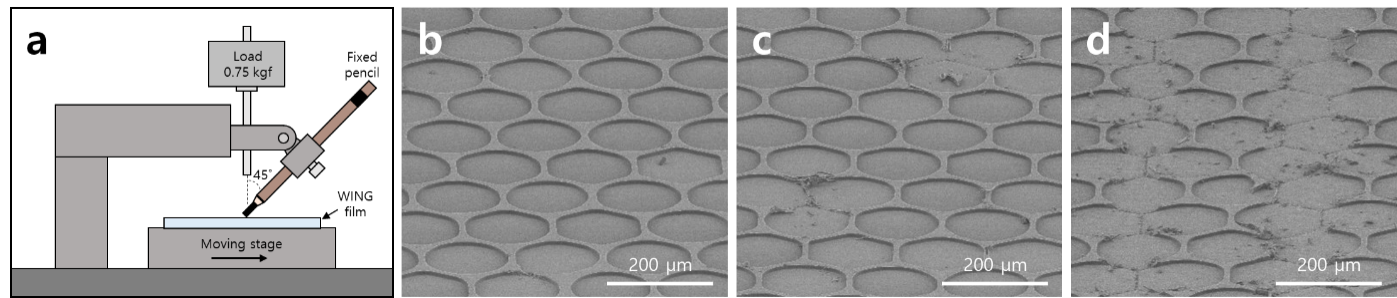


**Figure S1.** Pencil hardness test for mechanical stability of WING structures. (a) schematics of pencil hardness test. SEM images of WING structure after pencil hardness test. (b) 2 H, (c) 3 H, (d) 4 H.

Supporting Information 2. Effect of the surface energy of the resin-coated substrate on WING formation

**Figure S2a** shows that the surface energy of the resin-coated substrate is more than twice that of the resin and higher than the surface energy of the microstructure. It is assumed that the UV-curable resin is ideally coated with a uniform thickness. When the microstructure is in contact with the resin and separated after UV curing, the attractive force between the resin and coated substrate acts powerfully, and the cured resin is attached to the substrate via capillary action and is released. In Figure S2b, the surface energy increases in the following order: resin, substrate, and microstructure. A hyperbolic shape is formed owing to the capillary phenomenon that occurs when the resin is in contact with the microstructure, and a reentrant structure is ultimately formed through the UV-curing process. In Figure S2c, the surface energy increases in the following order: microstructure, UV-curable resin, and substrate. The resin on the substrate is coated in a droplet shape rather than a film of uniform thickness. This is because the surface tension of the resin is greater than the interfacial energy between the substrate and the resin. Furthermore, the resin is coated similarly, even when spray-coating technology is used. According to the second assumption shown in Figure S2b, capillary action occurs when the microstructure contacts the resin-coated surface. When the substrate is released after UV curing, a reentrant structure is obtained because the interfacial energy between the substrate and the cured resin is lower than that between the microstructure and the cured resin.


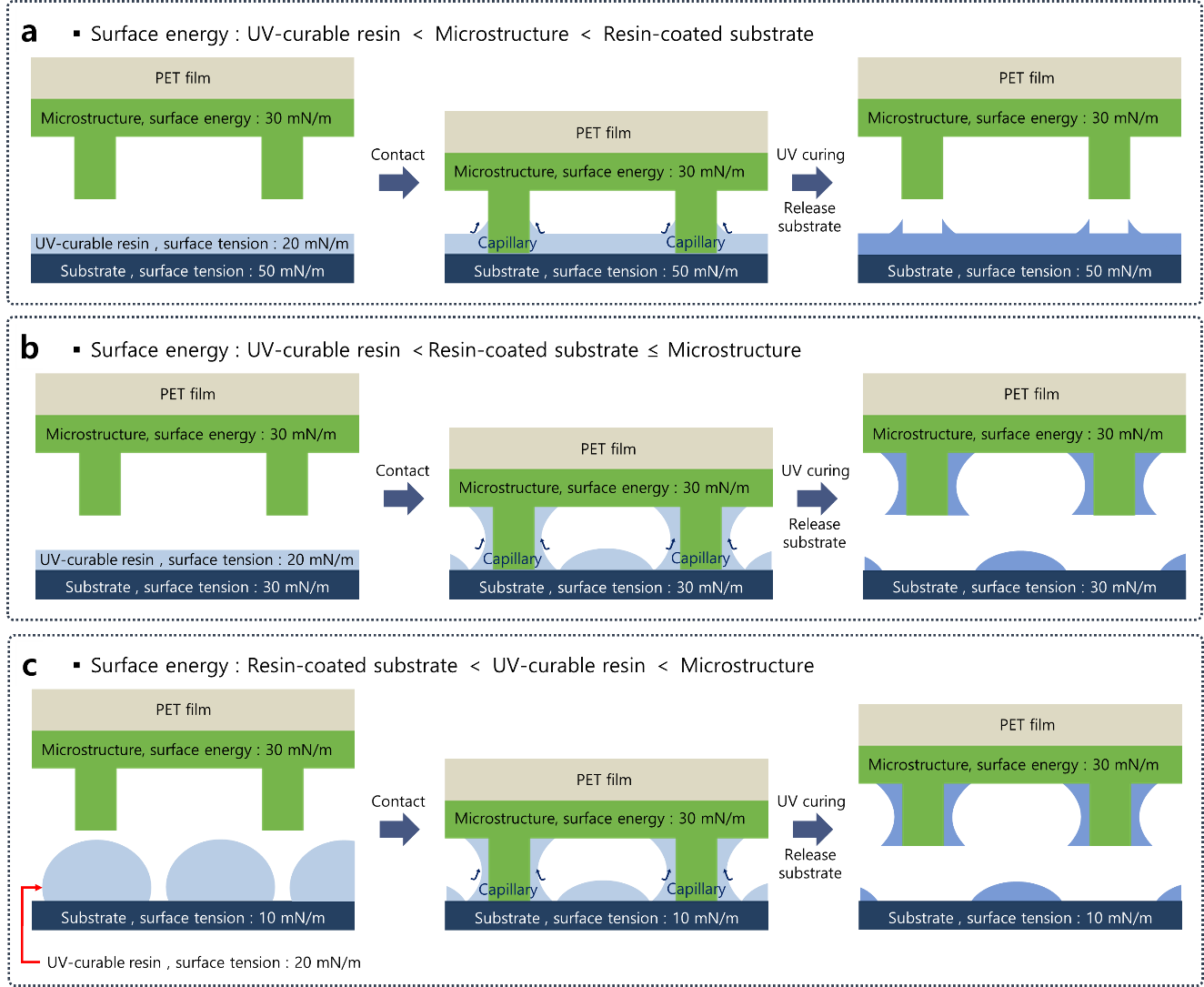


**Figure S2.** Assumptions of reentrant microcavity structure formation according to surface energy of the resin-coated substrates. (a, b) Ideal case, where the resin is coated with a uniform thickness; (c) the resin is coated in droplet form through spray-coating technology.

Experiments were conducted to verify this assumption, and the results are shown in **Figure S3**. Figure S3(a–c) indicate that the surface energy of the resin-coated substrate is higher than those of the microstructure and coated resin, as shown in Figure S2a. The experimental results were consistent with these assumptions. As described above, the capillary action of the coated resin occurs along the side of the microstructure to form a hyperbolic reentrant structure. However, in the release process after UV curing, strong adhesion occurred at the interface between the substrate and the resin with a high surface energy, resulting in the fabrication of the surface structure shown in Figure S3c. The experimental results obtained when the surface energy of the substrate was lower than those of the microstructure and resin are shown in Figure S3d–f. The results of this experiment were confirmed by the results shown in Figure S2c. Capillary action forms hyperbolic reentrant structures. Strong adhesion occurs at the interface between the resin and the microstructure according to the surface energy correlation at each interface during the release process after UV curing, and a reentrant structure is finally formed.


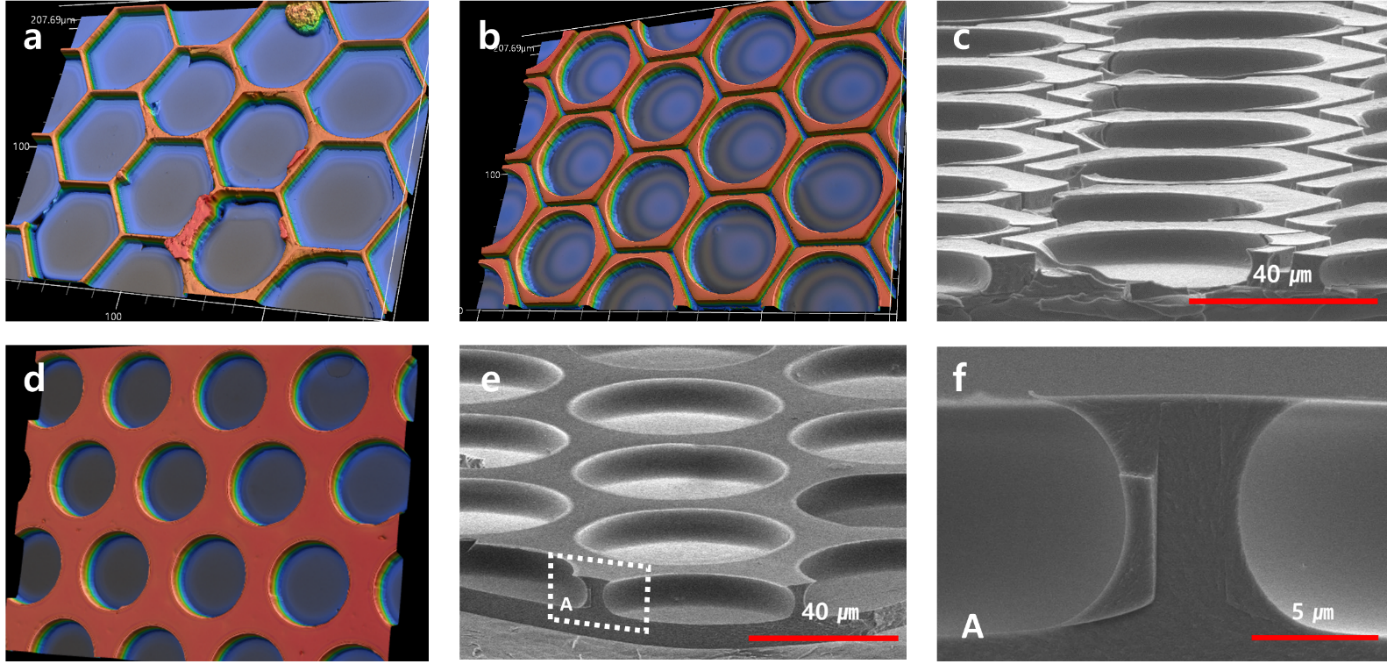


**Figure S3.** Experimental results for verification of assumptions on reentrant-structure formation: (a–c) when the surface energy of the resin-coated substrate is the highest; (a) 3D laser scanning image of the microstructures after the WING process; (b) 3D laser scanning image of the resin-coated substrate after the WING process; (c) SEM image of the resin-coated substrate after the WING process; (d–f) when the surface energy of the resin-coated substrate is the lowest; (d) 3D laser scanning image of the reentrant surfaces after the WING process; (e, f) cross-sectional SEM images of reentrant structures.

Supporting Information 3. Effect of the resin coating thickness on the Reentrant structure formation

The experiment was conducted to evaluate the significant effect of resin coating thickness on the WING process and to identify the maximum thickness required for the effective fabrication of the WING structure. This is crucial because the shape of the WING structure varies depending on the resin coating thickness, and if the interconnected microstructure comes into contact with an excessively thick resin layer, the microcavities may become filled, preventing the formation of the WING structure. In the experiment, the resin was sprayed onto a substrate with attached Kapton tape, followed by UV irradiation to pre-cure the resin. To measure the resin coating thickness, the resin was applied in a thin film rather than in droplet form. Complete curing was avoided, as the UV-curable resin experiences shrinkage during curing, which would affect the accurate measurement of the coating thickness. The Kapton tape was then removed to create an uncoated area (**Figure S4a**).

A thickness variation occurred between the center and the outer parts of the coated resin surface along the length of the removed tape, as illustrated in the cross-sectional schematic in Figure S4b. Based on this observation, spray coating was repeated 3, 5, and 10 times under the same conditions, and the coating thickness at both the center and a point 30 mm away from the center was measured. The WING process was subsequently carried out under the same conditions, and the resulting WING structures were analyzed based on the resin coating thickness.

The maximum resin coating thickness required for the WING process was determined. The coating thickness was measured using 3D laser scanning, distinguishing between the center and outer regions of the coated area, as shown in Figure S4c. It was confirmed that the WING structure formed successfully when the resin coating thickness was less than 6 μm. The measured coating thicknesses and the surface morphologies of the reentrant structures are presented in **Table S1** and **Figure S5**. A cross-sectional SEM image of the reentrant structure fabricated through the WING process with a resin coating thickness below 6 μm is shown in **Figure S6**.


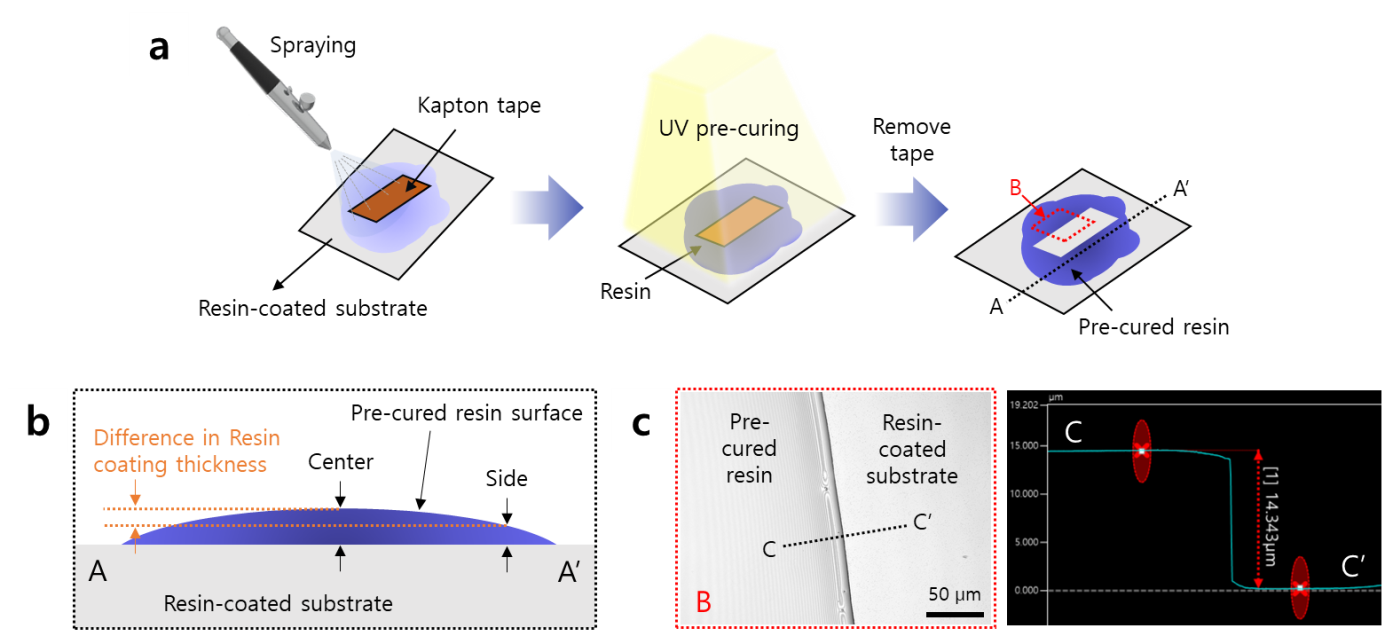


**Figure S4**. (a) Measurement method for the resin coating thickness. (b) Difference in thickness between the center and the side of the resin coated in a thin film form using the spray coating technique. (c) 3D laser scanning images of center area for measuring resin coating thickness.

**Table S1.** Measurement results for the resin coating thickness.

| Number of spray coatings | Resin coating thickness (µm) | | |
| --- | --- | --- | --- |
|  | Center | Side |  |
| 3 | 13.0 ± 2.8 | 3.3 ± 1.5 |  |
| 5 | 14.2 ± 0.2 | 4.4 ± 2.4 |  |
| 10 | 14.8 ± 0.3 | 5.7 ± 1.0 |  |


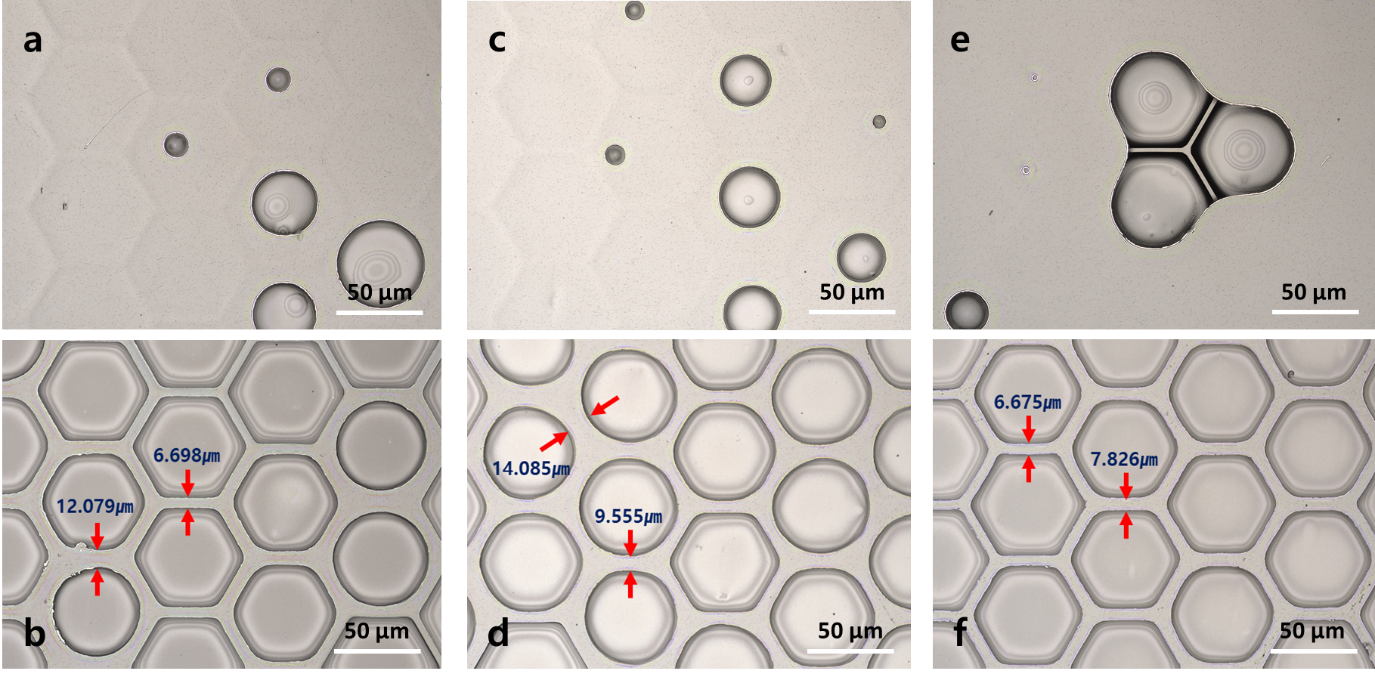


**Figure S5.** Experimental results for confirmation of the effects of the resin coating thickness on reentrant-structure formation. Number of spray coatings: (a, b) 3; (c, d) 5; (e, f) 10. (a, c, e) 3D laser scanning images of the central part; (b, d, f) 3D laser scanning images of the side part.


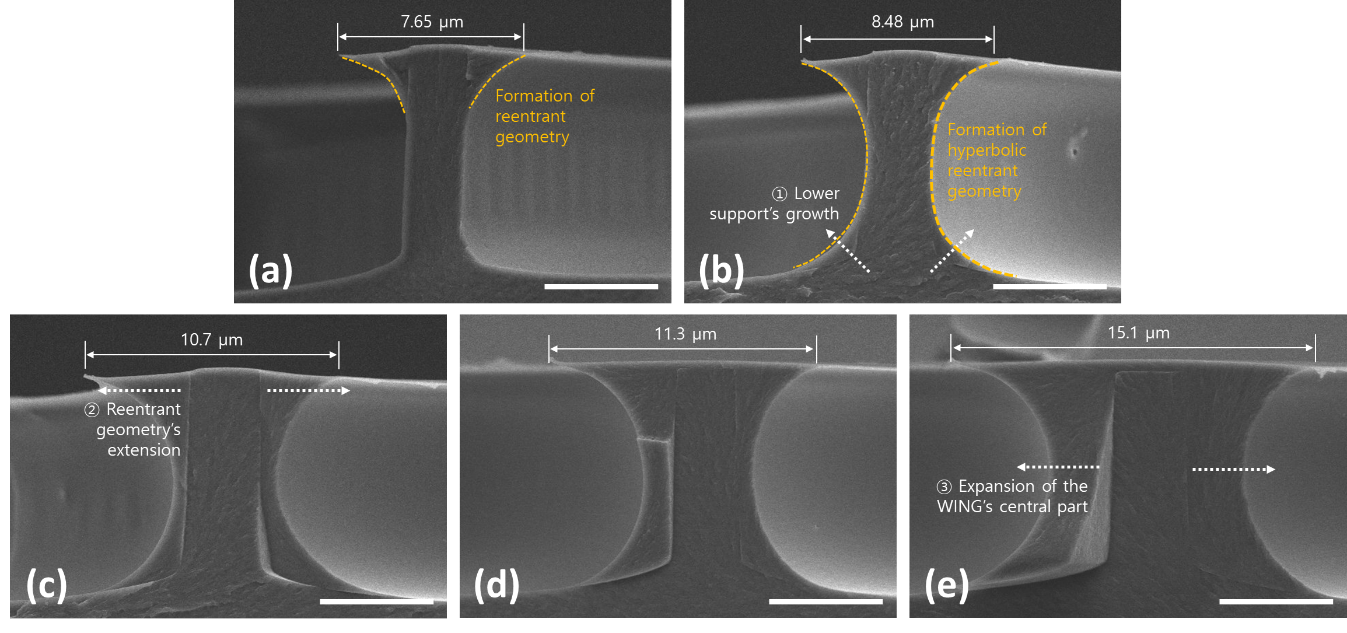


**Figure S6.** Comparison of WING structures formed according to resin coating thickness; Resin coating thickness: (a) 1.6 μm (± 0.2 μm), (b) 2.7 μm (± 0.5 μm), (c) 4.7 μm (± 0.2 μm), (d) 5.8 μm (± 0.3 μm), (e) 8.5 μm (± 0.6 μm). The white scale bars represent 5 μm.

Supporting Information 4. Experiment on the influence of microstructure geometry in manufacturing reentrant structure through WING process

The shape of the microstructure used in the WING process can influence the reentrant geometry, resulting in variations in the loading performance of different materials. To investigate the effect of microstructure geometry, an experiment was conducted. By varying the length of one side of the hexagonal pattern, as shown in **Figure S7a**, four different hexagonal reentrant microcavity structures were fabricated, and the solid fraction (*f_sl_*) was calculated to assess water repellency. The experiment utilized hexagonal reentrant microcavity structures with sidelengths of 40, 60, 80, and 100 μm, as shown in Figure S7(b–e).

Furthermore, the solid fraction is a key parameter for analyzing the liquid repellency of surfaces with microstructures. It represents the ratio of the solid surface in contact with the droplet on the microstructured surface and can be calculated using **Equation S1**.

$f_{sl}=\frac{A_{sl}}{A_{c}}$ (S1)

Here, *A_sl_* represents the actual contact area of the liquid droplet on the solid surface when it interacts with the microstructured surface, and *A_c_* represents the area projected from the top view. Therefore, the solid fraction (*f_sl_*) of the hexagonal pattern can be calculated using the area of the hexagon, which is the unit cell of the microstructure, and the surface area of the inner cavity, which is circular, as shown in Figure S7f. By substituting the calculated *f_sl_* and the intrinsic contact angle for water on the UV-curable resin used in the experiment, *θ_0_* = 80˚, into **Equation S2**, the theoretical contact angle (*θ_A_*) for water on the hexagonal reentrant microcavity structure can be determined.

$cos\theta_{A}=f_{sl}\left( cos\theta_{0}+1 \right)-1$ (S2)

The theoretical contact angle calculated in this manner differs slightly from the experimentally measured contact angle, but both show a trend of increasing as the solid fraction decreases. The discrepancy between the theoretical and experimental contact angles is likely due to the air pocket effect, caused by air trapped inside the microcavities, which is not accounted for in the theoretical calculation.

Based on these experimental results, the WING process described in this manuscript was applied to a hexagonal pattern with a sidelength of 100 μm. This pattern, with its low solid fraction and wide microcavities, provides favorable conditions for multi-material loading.


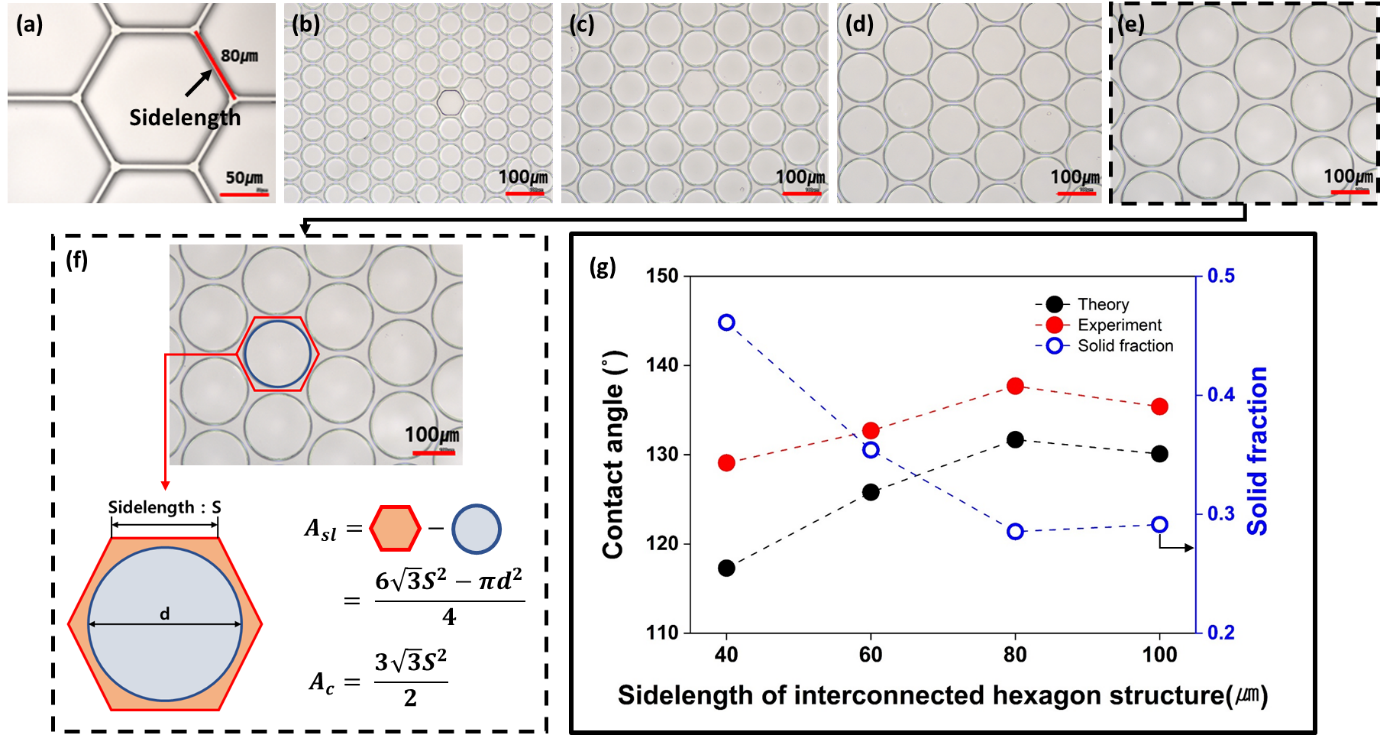


**Figure S7**. (a–e) 3D laser scanning images. (a) Interconnected hexagonal shape pattern (sidelength: 80 μm). (b–e) Hexagonal interconnected reentrant microcavity structures (sidelength: 40, 60, 80, 100 μm). (f) Calculation method of the solid fraction on the WING surfaces. (g) Liquid-repellency of the WING surface formed with the sidelength of the interconnected hexagonal pattern as a variable and the trend of the solid fraction.

Supporting Information 5. Design of the multistep R2R WING process system and developed process equipment (film width: 1200 mm)

A schematic of the multistep R2R WING process is shown in **Figure S8**, which is primarily composed of two steps: the microstructure is first patterned using R2R UV nanoimprint lithography, and then the WING process is applied to manufacture a large-area reentrant-structured film. Compared with the existing R2R nanoimprint lithography process, in addition to the unwinding and rewinding system that transports and recovers the microstructure film, a separate unwinding and rewinding system is needed to transport and recover the release film spray-coated with UV-curing resin. The two winding systems maintain the tension of the film engraved with the microstructure and release film coated with the resin. Consequently, a release force occurs between the two films after the curing process is complete, and a multifunctional reentrant microcavity film is manufactured.

**
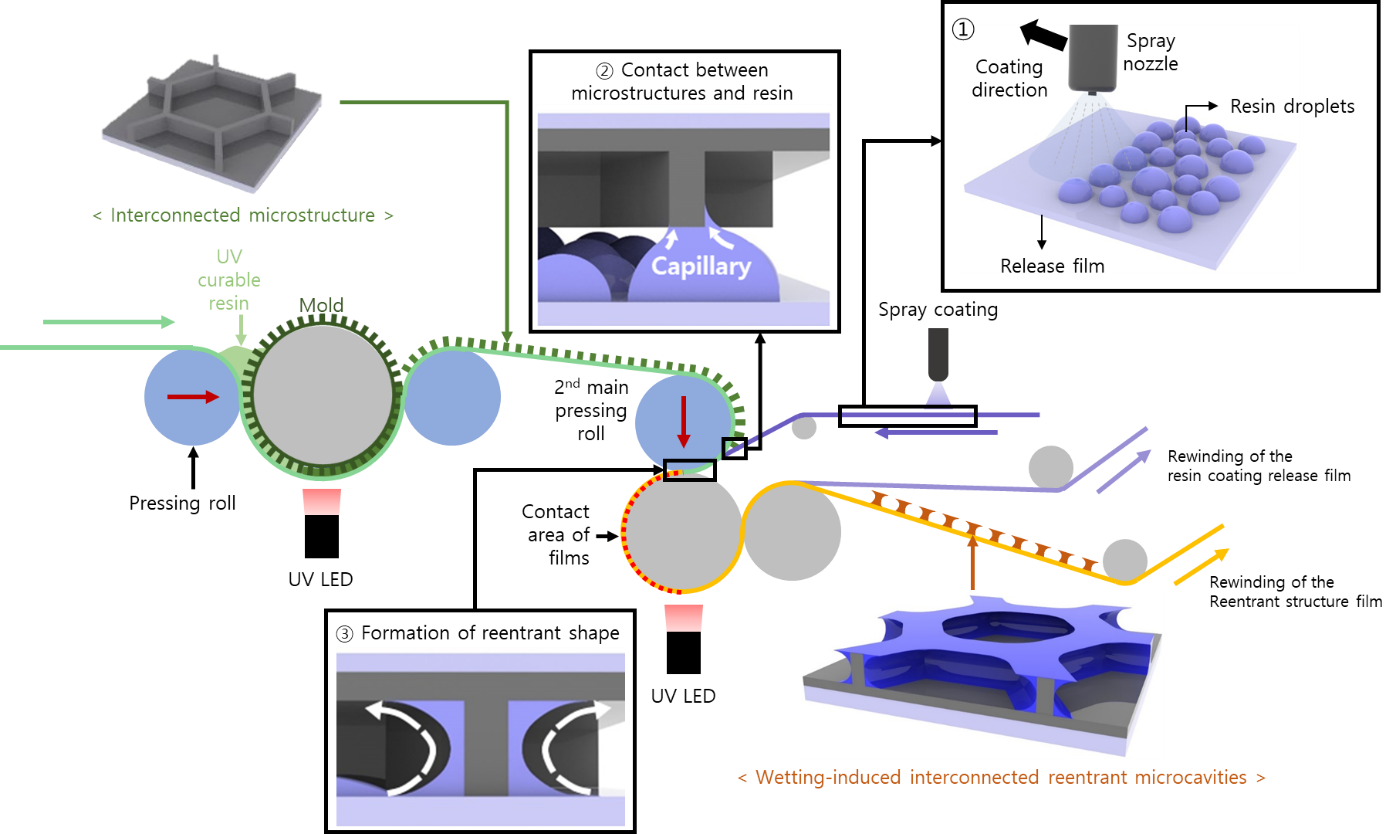
**

**Figure S8.** Schematic of the multistep R2R WING process.

The developed process equipment is shown in **Figure S9**. The main components are an unwinder and rewinder for transfer and tension control of the PET web film; the 1^st^ UV imprint module, which performs the UV-NIL process; the 2^nd^ WING module, which forms a reentrant structure by laminating the microstructure-engraved film and resin-coated release film; and a buffer roll system to prevent defects such as slip that can occur when the film is in contact with the 1^st^ and 2^nd^ process modules.

**
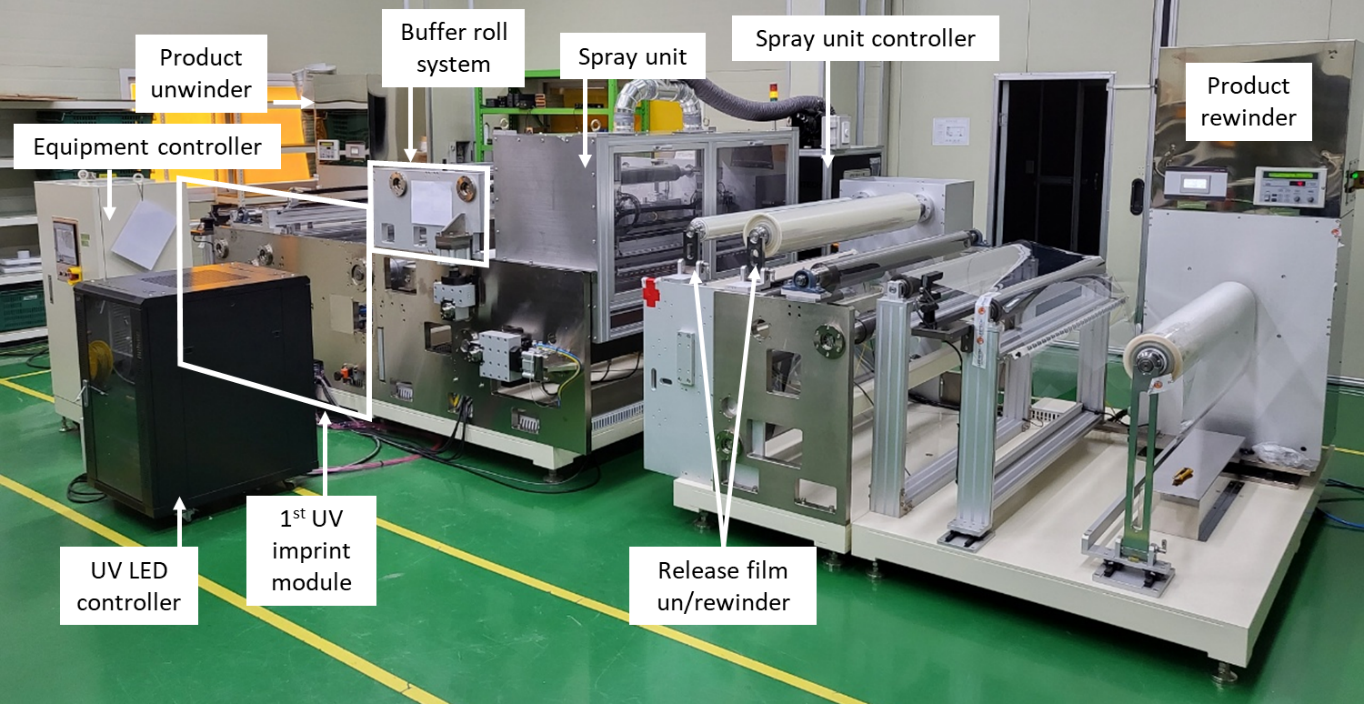
**

**Figure S9.** Developed multistep R2R WING process system for a film width of 1200 mm.

Supporting Information 6. Optimization of R2R WING process for manufacturing large-area surfaces with uniform reentrant structures.

To pattern a uniform WING structure on a large-area film with a width of 1,200 mm using the R2R WING process, process optimization experiments were conducted, beginning with confirming the resin spray coating thickness. As mentioned in Supporting Information 2, the maximum allowable resin coating thickness for forming the WING structure is 6 μm. Accordingly, experiments were performed to verify the resin coating thickness under different spraying conditions, using the system developed for the R2R WING process, as shown in Figure S9. The spray pressure was set as a variable, with the conditions outlined in **Table S2**. The experiment involved supplying a PET web film that had not undergone the 1^st^ UV nanoimprint lithography process, as shown in **Figure S10a**, and preparing samples for measuring the resin coating thickness. The resin was cured using the UV LED positioned at the bottom after the PET film had come into contact with the release film coated via spray coating. At this stage, no load was applied to the 2^nd^ pressure roll located above the two films in contact. The results confirmed that a uniform resin coating layer with a thickness of 6 μm or less was achieved under spray pressure conditions of 350 kPa or higher (Figure S10b).

**Table S2.** Conditions of resin coating thickness confirmation experiments with spray pressure as a variable.

| UV power [mW/cm^2^] | 570 | | | | |
| --- | --- | --- | --- | --- | --- |
| Web film feed rate [mm/s] | 20 | | | | |
| Spray nozzle speed [mm/s] | 300 | | | | |
| Supply amount of resin [ml/min] | 0.5 | | | | |
| Spray pressure [kPa] | 200 | 250 | 300 | 350 | 400 |


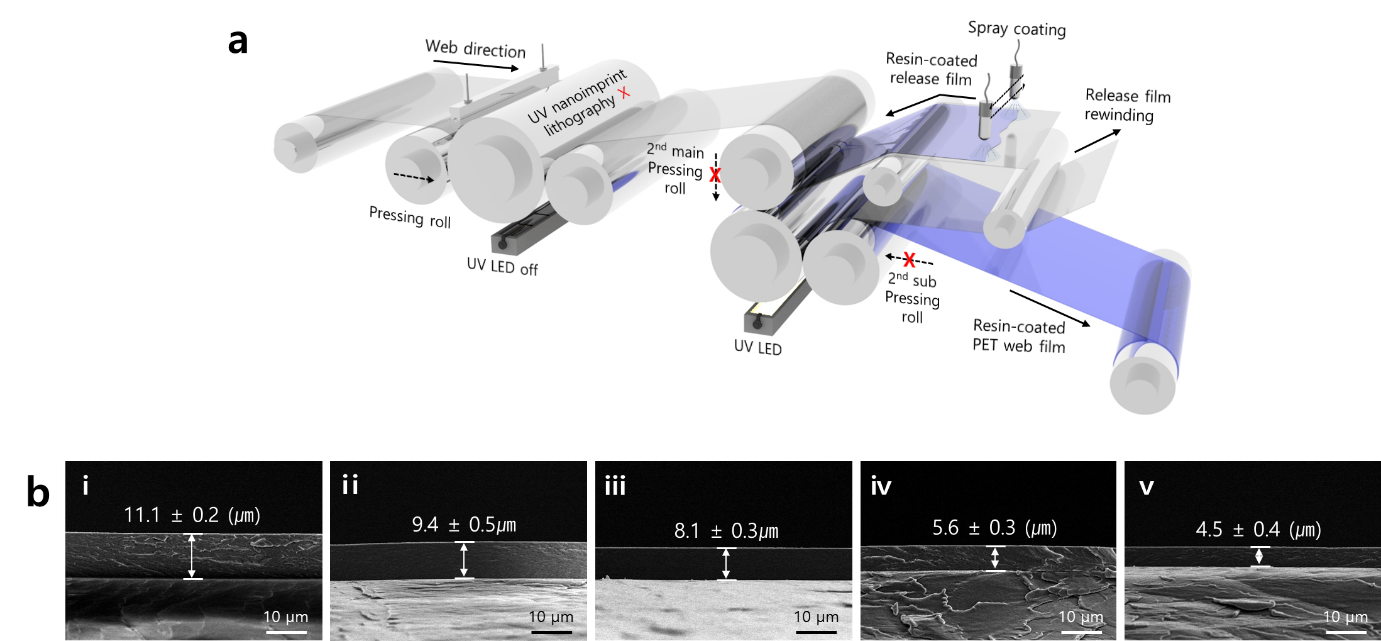


**Figure S10.** (a) Schematic of the confirmation experiment via R2R WING process system. (b) SEM cross-sectional images of resin coating films formed by spray pressure conditions. (ⅰ) 200 kPa, (ⅱ) 250 kPa, (ⅲ) 300 kPa, (ⅳ) 350 kPa, (ⅴ) 400 kPa.

Using the resin coating conditions mentioned above, an experiment was conducted to form a uniform WING structure on the surface of a 1,200 mm wide roll film via the R2R WING process. The main variables in the experiment were the resin supply for spray coating, spray pressure, and roll pressing force (**Table S3**). The resin supply and spray pressure were selected as variables because, under specific spray pressure conditions, an increased resin supply can lead to a thicker or uneven coating. Additionally, the contact state between the films changes depending on the pressing force applied when the two films come into contact, affecting the uniformity of the WING structure. Therefore, pressing force was also selected as a variable.

To analyze the uniformity of the WING structure formed on the large-area film, 50 × 50 mm samples were collected from the center, left, and right positions of a 1,000 mm wide film, and the uniformity of the upper width of the WING structure was compared (**Figure S11**). An actual image of the obtained sample is shown in **Figure S12**. In the results of experiment #1, where no force was applied to the 2^nd^ main pressing roll (Figure S12), the surface was found to be non-uniform, likely due to inconsistent contact between the two films. However, in the other experiments where force was applied to the 2nd pressing roll, samples with relatively uniform surfaces were obtained.

A comparison of the uniformity of the upper width of the WING structure across the samples is presented in **Figure S13**. The results indicate that the resin supply has a greater influence on the uniform patterning of the WING structure on a large-area film than roll pressure. In particular, when the resin supply was increased and appropriate spray pressure conditions were applied, it was possible to fabricate a WING structure with an upper width variation within ± 2 μm.

**Table S3.** Conditions of the experiments to secure uniformity of the WING structure fabricated through R2R WING process.

| Number of experiments | 1 | | 2 | | 3 | | 4 | | 5 |
| --- | --- | --- | --- | --- | --- | --- | --- | --- | --- |
| UV power [mW/cm^2^] | 570 | | | | | | | | |
| Web film feed rate [mm/s] | 20 | | | | | | | | |
| Spray nozzle speed [mm/s] | 300 | | | | | | | | |
| 2^nd^ main pressing roll force [kgf] | 0 | 190 | | 380 | | 190 | | 190 | |
| Supply amount of resin [ml/min] | 0.5 | 0.5 | | 0.5 | | 1.5 | | 1.5 | |
| Spray pressure [kPa] | 400 | 400 | | 400 | | 400 | | 300 | |


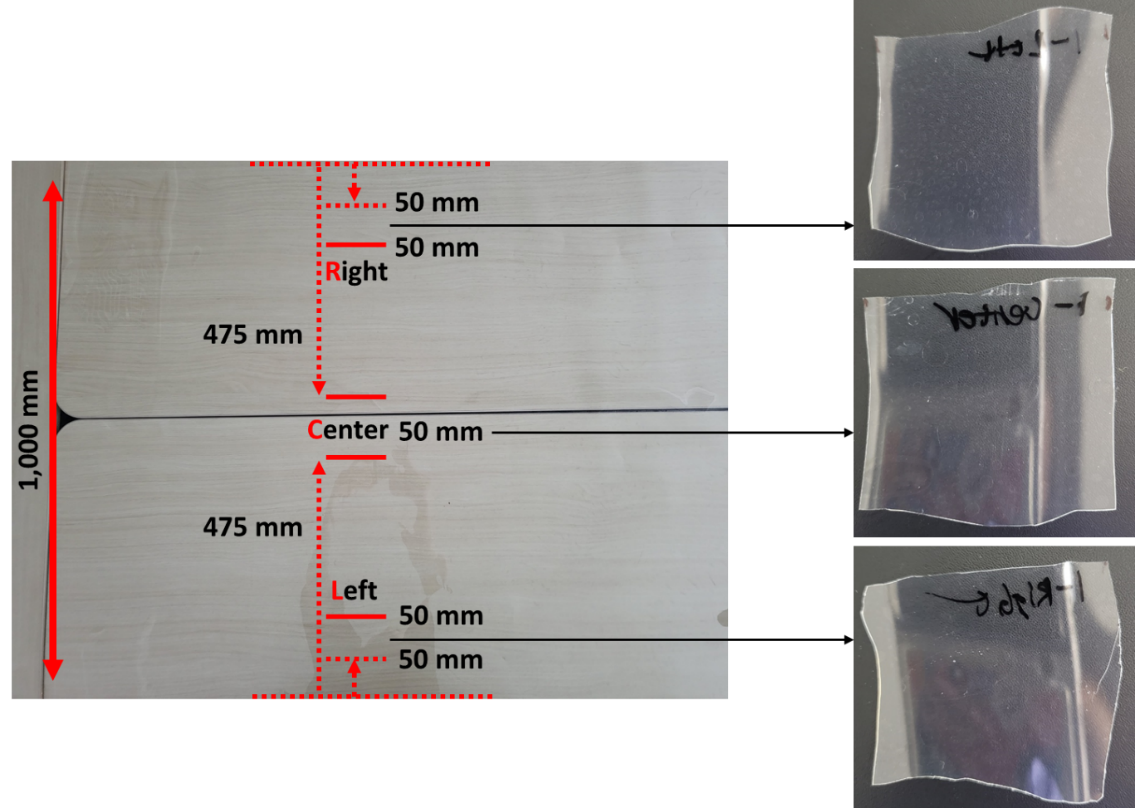


**Figure S11.** Sample collection positions for confirmation of the uniformity of the fabricated WING structure.


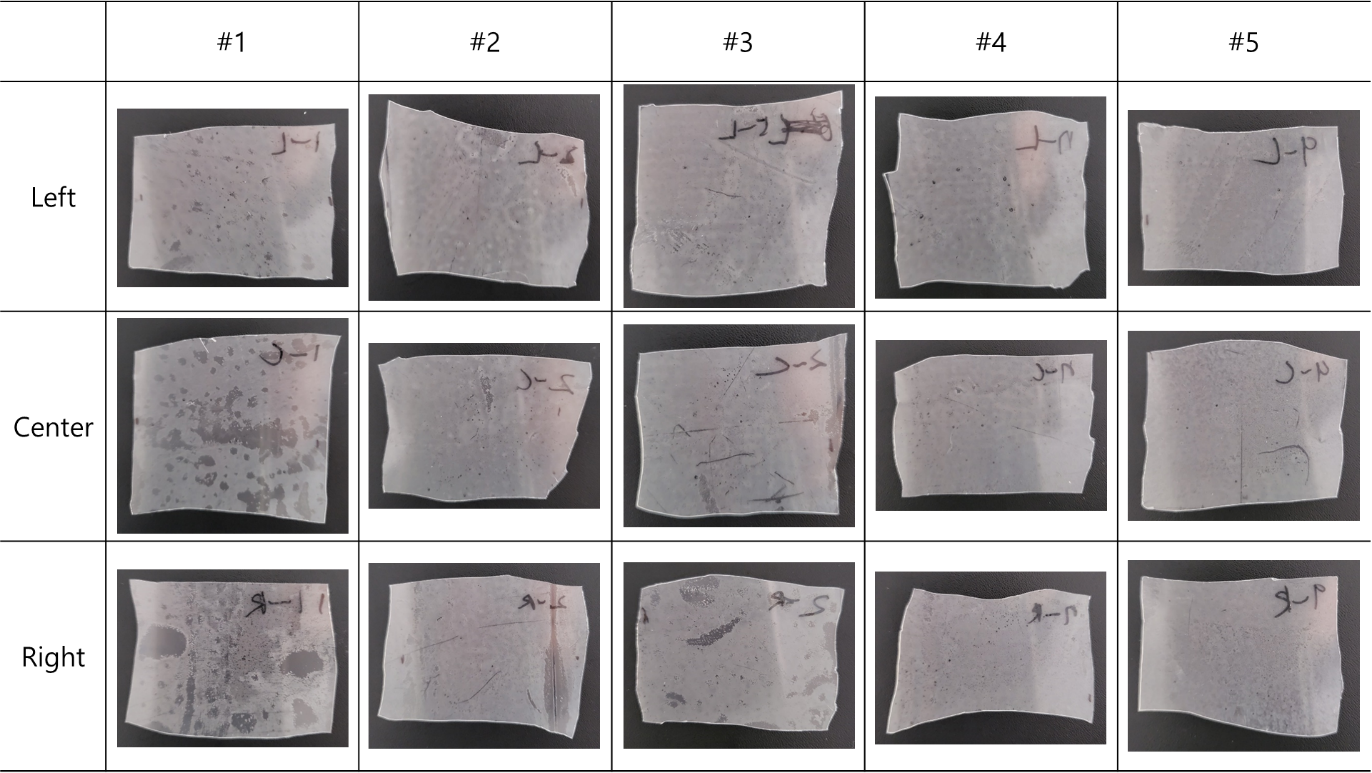


**Figure S12.** Actual photos of samples fabricated as a result of the R2R WING process optimization experiments.


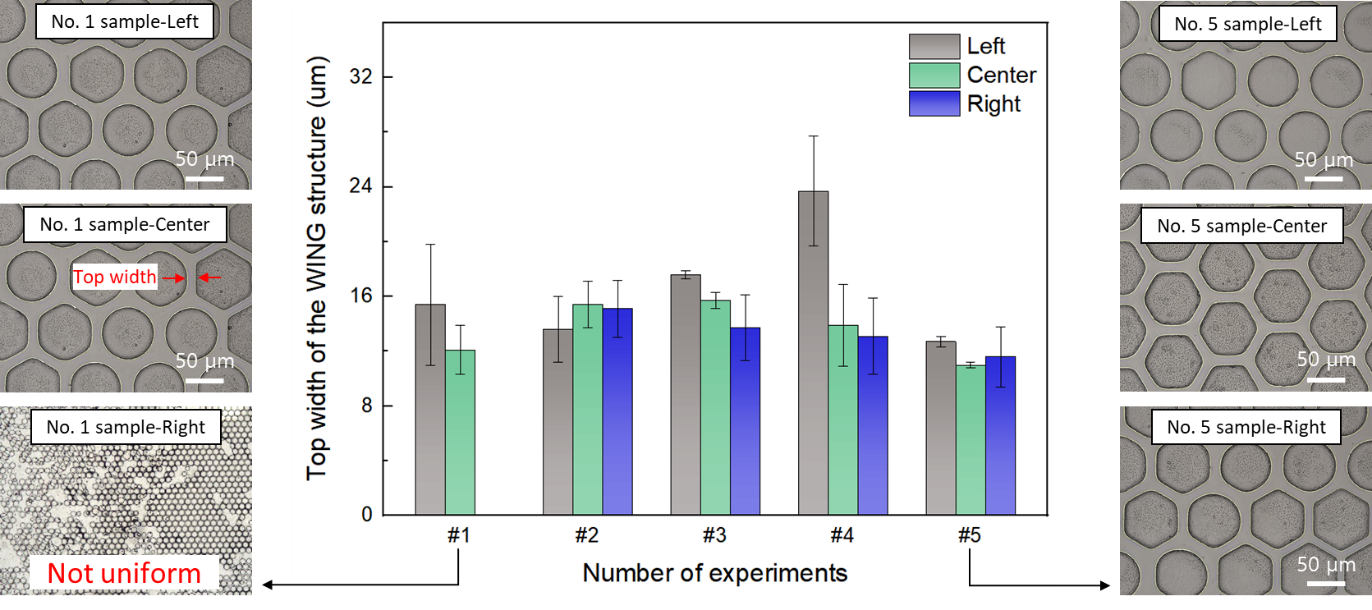


**Figure S13.** R2R WING process optimization experiment results for fabrication of uniform WING structure on the large area film with width 1,200 mm.

Supporting Information 7. Mechanism of capillary action that occurs during the WING process

A dimensionless coefficient called the capillary number was introduced to analyze the mechanism of capillary action during the WING process. The capillary number is a dimensionless coefficient that represents the relative effect of the viscous force on the interfacial energy between the liquid and surface. According to previous research on capillary number, if the capillary number is larger than 10^-4^, the viscous force has a dominant influence on the behavior of the fluid, and if it is small, the capillary force can be interpreted as having a dominant influence.^[43]^ The capillary number can be calculated using Equation S3 and is expressed as a formula for the viscosity (*η*), liquid surface tension (*γ_polymer/air_*), and resin contact angle (*θ_polymer/solid_*) on the microstructured surface.

$Ca=\frac{\eta V}{\gamma}=\eta\sqrt{\frac{2cos\theta_{polymer/solid}}{\gamma_{polymer/air}\rho R}}$ (S3)

Here, *Ca* represents the capillary number, *V* represents the capillary velocity, and *ρ* represents the resin density. *R* represents the hydraulic radius, for which half of the microstructure height, i.e., the height of the capillary rise, is substituted. The capillary numbers calculated using the physical properties of each resin exceeded 10^-4^, as shown in Table S4; thus, it was confirmed that the WING process was performed under the influence of a viscous force.

**Table S4.** The capillary number was calculated to analyze the effect of the resin viscosity on the capillary action at the time of reentrant-structure formation.

| Coated resin | Viscosity  ($\eta$, cPs) | Contact angle  ($\theta$, ˚) | Surface tension  ($\gamma$, mN/m) | Density  ($\rho$, g/cm^3^) | Hydraulic radius  ($R$, µm) | Capillary number ($Ca)$ |
| --- | --- | --- | --- | --- | --- | --- |
| A | 22 | 23.0 | 19.2 | 1.06 | 4.5 | 3.1 |
| B | 34 | 21.9 | 19.1 | 1.08 |  | 4.8 |
| C | 56 | 22.5 | 17.0 | 1.09 |  | 8.3 |
| D | 106 | 22.9 | 18.5 | 1.10 |  | 15.0 |


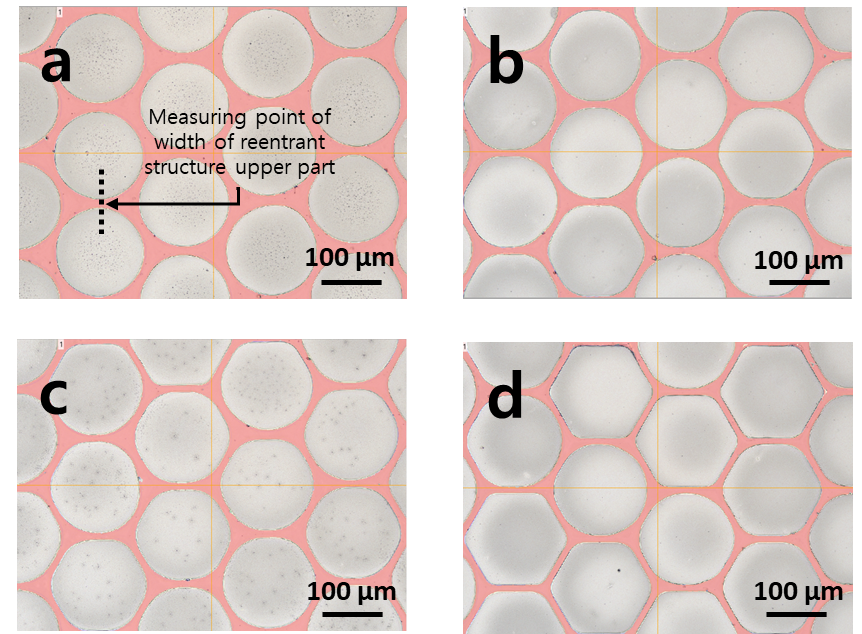


**Figure S14.** 3D laser scanning top view images of reentrant microcavities fabricated using UV-curable resins with various viscosities; (a) 106.2 cPs, (b) 55.8 cPs, (c) 34.1 cPs, (d) 22.3 cPs. (Surface color is highlighted to compare the surface morphology changes of reentrant microcavities.)

To geometrically analyze the shape of the WING structure, which varies according to the capillary meniscus formed during the WING process, Washburn's equation (Equation 1) and Equations 2–4 were derived using the simplified model shown in **Figure S15**. The derivation process for Equations 2–4 is explained in detail below.

In Equation 2, *V_ini_* represents the volume of the UV-curable resin coated at the microcavity location within the microstructure, as shown in Figure S15(i). This can be expressed by Equation 3, using the formula for calculating the volume of a cylinder with a thickness of *t_c_*. After the capillary meniscus forms, *V_aft_*, the volume of the UV-curable resin, can be obtained by subtracting the cone volume (*V_2_*) and the cylinder volume (*V_3_*) from the volume of the initial cylinder (*V_1_*), which has a diameter *D* and a height equal to the capillary rise height *z*, as depicted in Figure S15(ii). The process is outlined as follows.

$V_{1}=\frac{\pi}{4}\cdot D^{2}\cdot z(t)$ (S4)

$V_{2}=\frac{\pi}{3}\cdot{w(t)}^{2}\cdot z(t)$ (S5)

$V_{3}=\pi[\frac{D}{2}-{w(t)]}^{2}\cdot z(t)$ (S6)

$V_{1}-V_{2}-V_{3}=V_{aft}=\pi\cdot z(t)[D\cdot w(t)-\frac{4}{3}{w\left( t \right)}^{2}]$ (S7)

Assuming that Vini from Equation 2 and *V_aft_* from Equation 3 are equal and eliminating the *π* term, the equation can be rearranged into **Equation S8** below. The constant α introduced in Equation S8 represents the loss factor for the residual resin volume remaining in the center of the microcavity during capillary meniscus formation, as described in the manuscript. Therefore, the constant *α* ranges from 0 to less than 1 (0 ≤ *α* < 1). (**Figure S16**). Substituting Equation 1, which represents *z*, into this yields Equation 4. Here, *t* represents the capillary time, which refers to a brief duration in the millisecond range, so a value of 0.01 ms was substituted.

$V_{ini}\approx V_{aft}=\alpha\cdot{\frac{D}{4}}^{2}\cdot t_{c}\approx z(t)\cdot[D\cdot w\left( t \right)-\frac{4}{3}{w\left( t \right)}^{2}]$ (S8)

$V_{ini}\approx V_{aft}=\alpha\cdot{\frac{D}{4}}^{2}\cdot t_{c}\approx({\frac{\gamma Dt}{2\eta})}^{1/2}\cdot[D\cdot w\left( t \right)-\frac{4}{3}{w\left( t \right)}^{2}]$ (S9)


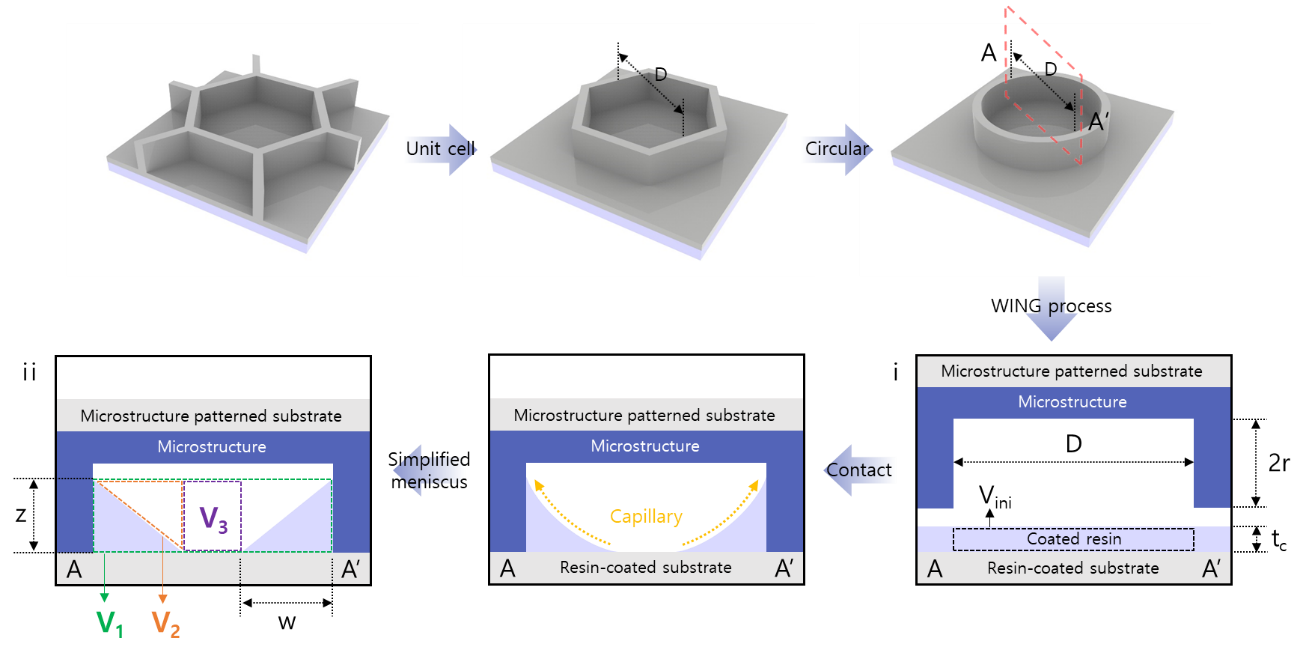


**Figure S15.** A simplified model for geometrical analysis of the WING structure fabricated according to the capillary meniscus formed during the WING process.


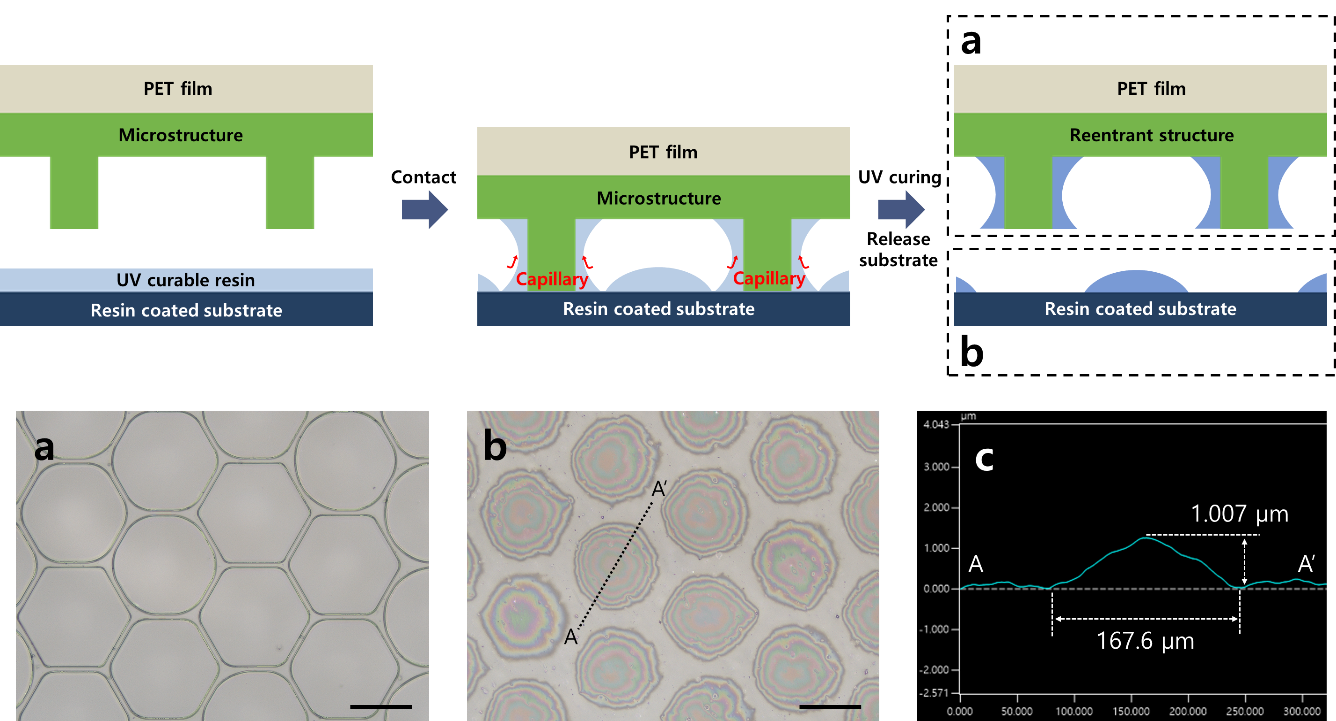


**Figure S16.** Aspects of the coated UV-curable resin after the WING process. 3D laser scanning images: (a) Reentrant structure formed on the microstructured surface, (b) residual resin on the resin coated substrate after the WING process, (c) cross-sectional profile of the residual resin.


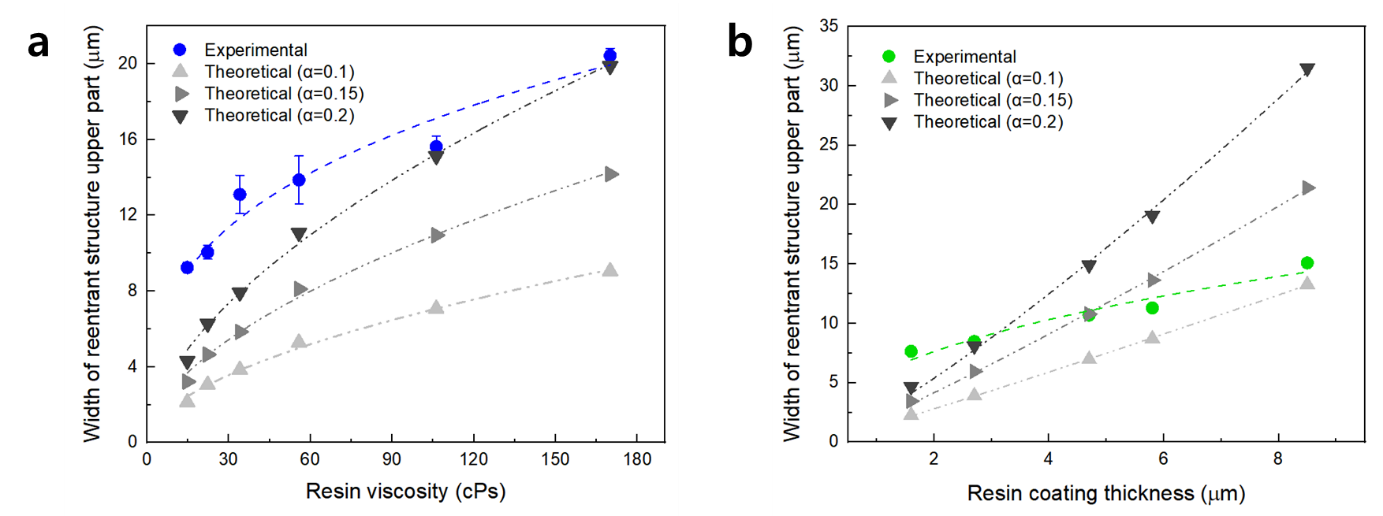


**Figure S17.** Geometrical analysis of the reentrant structure fabricated through the WING process under varying photocurable resin conditions. (a) Resin viscosity, (b) resin coating thickness.

Supporting Information 8. Silica nanoparticle coating for improving liquid-repellency of large-area WING film


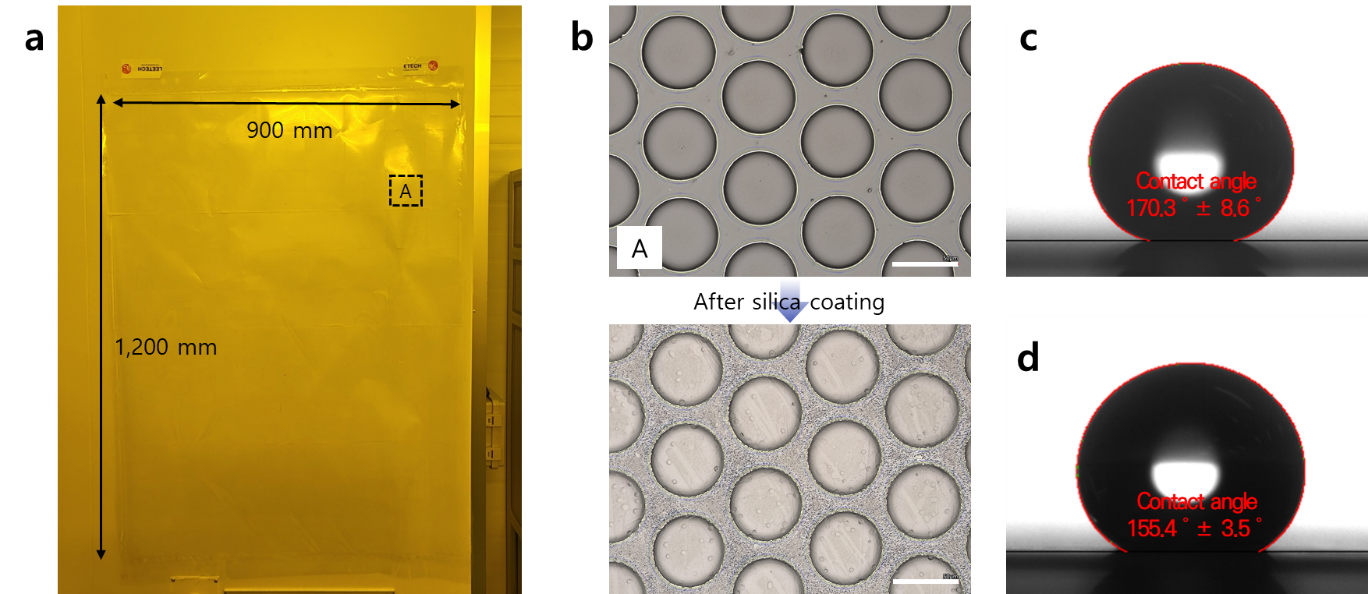


**Figure S18.** Silica nanoparticle coating for improved liquid repellency. (a) Large-area WING structure film coated with silica nanoparticles. (b) 3D laser scanning images of WING structure before and after silica nanoparticle coating. The white scale bars represent 50 μm. Contact angle measurement results using (c) DI water and (d) Olive oil.

**Table S5.** Comparison of the performances of different reentrant surfaces.

| Items | Hydrophobicity | Oleophobicity | Manufacturing area | Production speed  (Production time) | Robustness |
| --- | --- | --- | --- | --- | --- |
| Our research | 139.1˚ ± 1.6˚  (W. Si ^a)^ = 170.3˚) | 139.6˚ ± 2.6˚  (W. Si = 155.4˚, olive oil) | 1,200 mm  (Film width) | 20 mm/s  (Within 20 min) ^b)^ | 2.5 H (Pencil hardness test) |
| Ref. 17 | W. Si = 165˚ ± 1˚ | 154˚ (Tetradecane) | Several centimeters | More than  one day | 90×10^-8^ J/per grain  (Sand abrasion experiment) |
| Ref. 22 | W. Silane^c)^ = 149˚ | X | Several  centimeters | More than  two days | X |
| Ref. 4 | W. F^d)^ = 156˚ | W. F = 158˚  (Hexadecane) | 4 inch | about one day | -  (Only simulation data) |
| Ref. 6 | D. Al^e)^ = 161.9˚ | D. Al^e)^ = 156.4˚  (Vegetable oil) | 2 × 2 (cm^2^) | about 3 hour | Repeated cyclic stretching, bending, twisting test (> 1,000 cycles) |
| Ref. S1 | 161.3˚ | X | 300 mm  (Roll width) | 4.1 cm/s | Wear test with rubber tip,  ~ 2,000 cycles (Normal load: 1.5N) |
| Ref. S2 | 140˚  (W. F^d)^ = 155˚) | 83˚  (W. F^d)^ = 140˚, diiodomethane) | 1 × 1 (cm^2^)  (Microstructure mold) | 1.7 ~ 5 mm/s | X |
| Ref. S3 | X | X | 2 × 2 (cm^2^)  (Microstructure mold) | 1.7 mm/s | 13 N/cm^2^ (Pull-off strength) |
| Ref. S4 | W. F^d)^ = 160˚ | X | 6 inch  (Roll width) | 9 ~ 12 in/min | X |
| Ref. S5 | 160˚ | X | Several tens of centimeters | 333 mm/s | X |
| Ref. S6 | W. F^d)^ = >160˚ | X | 250 mm  (Film width) | 167 mm/s | X |

^a)^ With Silica coating; ^b)^ Manufactured film length: 1 m; ^c)^ Silanization treatment; ^d)^ With fluorine material; ^e)^ Deposition of a thin aluminum layer.

Supporting Information 9. Particle-loading within the reentrant structure

Particles were loaded onto the surfaces of microstructures by coating them with a solution containing particles dissolved in DI water at a specific concentration until the surfaces were sufficiently wetted, followed by drying to facilitate particle attachment. The mechanism of loading particles into microstructures by drying an aqueous solution can be understood through the principle of evaporation. Evaporation occurs when the kinetic energy of molecules surpasses the intermolecular forces, such as van der Waals forces, acting between the molecules on the surface of the water, as illustrated in **Figure S19a**. As a result, water molecules vaporize in the form of water vapor, and evaporation always occurs at the water's surface.

When this process is applied to a surface with an engraved reentrant structure, as shown in Figure S17b, the open structure at the upper surface of the cavity allows evaporation to begin from the top of the microcavity when the structure is filled with the aqueous solution and heat is applied. As the water evaporates from the surface, the remaining water and dissolved particles migrate to the sidewalls and edges of the reentrant structure. Ultimately, the particles are distributed along the sidewalls and edges.

If the surface where the particles remain after evaporation lacks a microstructure (such as in Figure 4a and 4b) or has an open structure like a pillar structure, the particles can easily propagate to other places, as observed in the tape peel-off test results. However, the reentrant structure offers a structural advantage, with the upper part of the pattern protruding horizontally, preventing the particles from detaching.


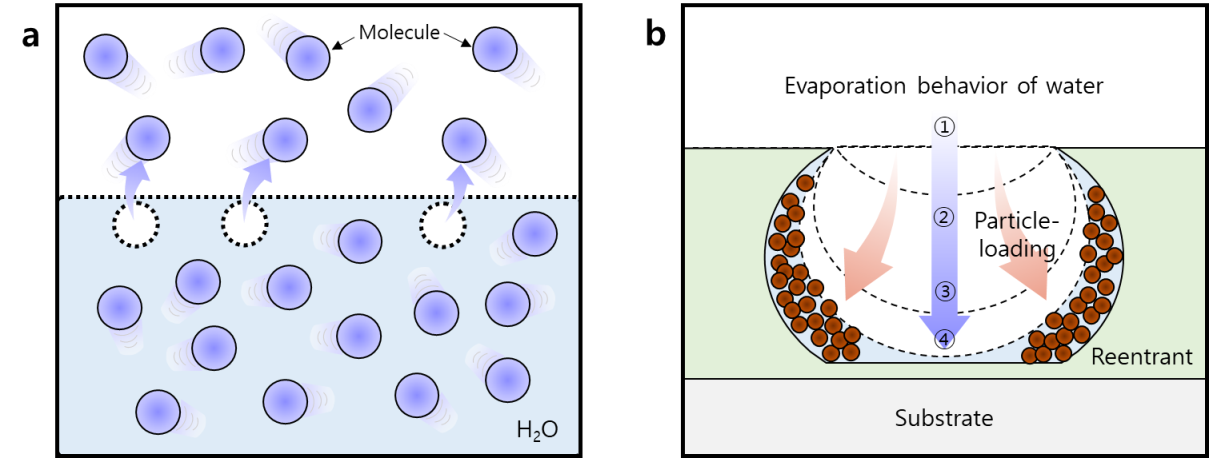


**Figure S19.** (a) Schematic diagram of the principle of water evaporation. (b) The process of loading particles as water evaporates from the reentrant microcavities within the reentrant structure immersed in the aqueous solution containing nanoparticles.

**Supporting Information 10. Designed interconnected microstructures**

**
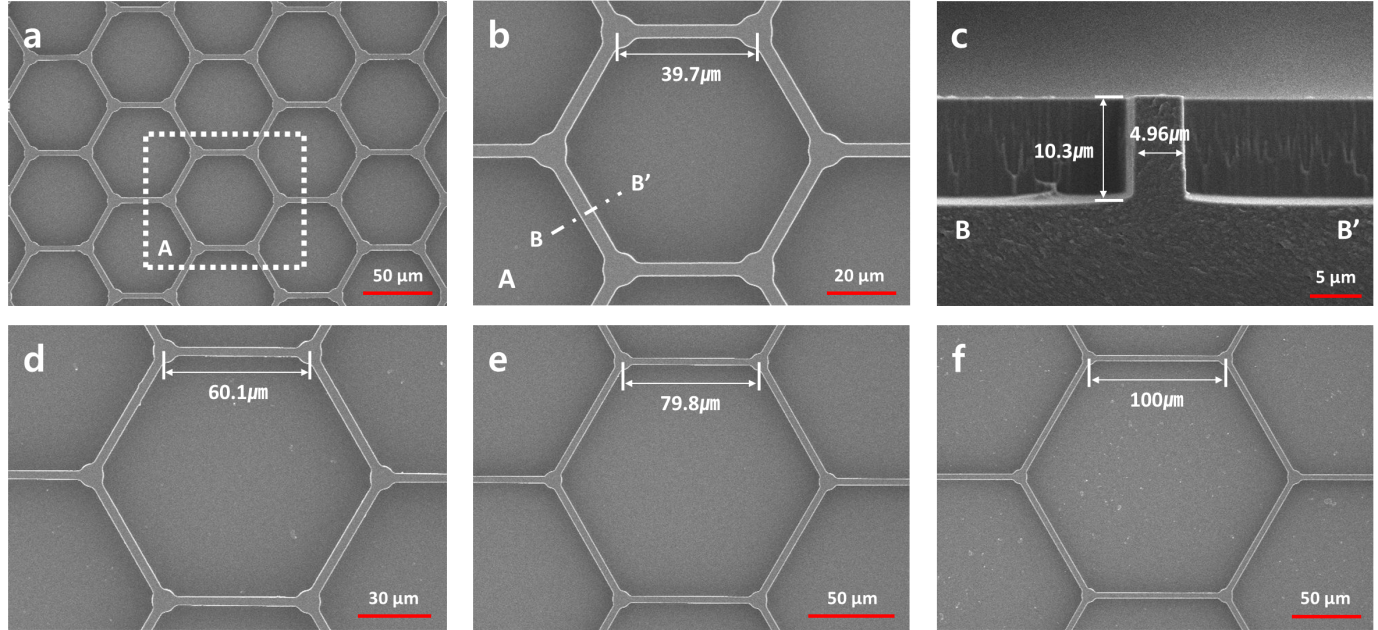
**

**Figure S20.** SEM images of the interconnected microstructures. (a–c) designed with sidelength 40 µm: (a, b) top view, (c) cross-sectional view. Top view designed with (d) 60 µm, (e) 80 µm, (f) 100 µm.

References

[S1] S. Park, S. Lee, D. Moreira, P. R. Bandaru, I. Han, D. Yun, Sci. Rep. **2015**, 5, 15430.

[S2] N. K. Shivaprakash, J. Zhang, A. Panwar, C. Barry, Q. Truong, J. Mead, J. Appl. Polym. Sci. **2019**, 136, 46980.

[S3] S. H. Lee, C. W. Park, M. K. Kwak, J. Chem. **2019**, 2019, 4827918.

[S4] Y. Li, J. John, K. W. Kolewe, J. D. Schiffman, K. R. Carter, ACS Appl. Mater. INTER. **2015**, 7, 23439–23444.

[S5] A. Telecka, S. Murthy, L. Schneider, H. Pranov, R. Taboryski, ACS Macro Lett. **2016**, 5, 1034–1038.

[S6] M. Leitgeb, D. Nees, S. Ruttloff, U. Palfinger, J. Götz, R. Liska, M. R. Belegratis, B. Stadlober, ACS nano **2016**, 10, 4626–4941.
